# Exercise impairment in patients with pectus excavatum? A scoping review of evidence and role of arterial content change during effort

**DOI:** 10.14814/phy2.71005

**Published:** 2026-07-06

**Authors:** Maxime Lokietek, Simon Lecoq, Laura Filaire, Bénédicte Noury‐Desvaux, Mariève Houle, Pierre Abraham

**Affiliations:** ^1^ Univ Angers, Inserm, CNRS, MITOVASC, Equipe CarMe, SFR ICAT, F‐49000 Angers France; ^2^ Sports Medicine Department Angers University Hospital Angers France; ^3^ Department of Thoracic Surgery University Hospital of Saint Etienne, North Hospital Saint‐Priest‐en‐Jarez France; ^4^ Institute of Physical Education and Sport Training Catholic University of the West Les Ponts‐de‐Cé France

**Keywords:** exercise test, funnel chest, hemoglobin, oxygen, physiology

## Abstract

This scoping review describes cardiopulmonary fitness in non‐operated patients with pectus excavatum and maps the reporting of hemoglobin and arterial oxygen content during exercise. Four databases (PubMed, ScienceDirect, CINAHL, and SPORTDiscus) were searched from inception to February 15th, 2025. Cross‐sectional studies, cohort studies, and randomized controlled trials reporting exercise capacity or exercise testing outcomes were included. Among 612 identified records, 31 studies involving 2937 patients met all eligibility criteria. Seventeen studies reported maximal oxygen uptake (VO_2_max) as a percentage of predicted values, ranging from 62% ± 25% to 101.0% ± 18.3%, indicating substantial heterogeneity in exercise capacity. Hemoglobin concentration was quantified in only one study (11.6 to 17.3 g/100 mL). Pulse oximetry was assessed in 15 studies but quantitatively reported in four (95.5% ± 2.1% to 97.4% ± 1.2%). Blood gases were assessed in five studies, with results reported in two, including alveolar–arterial oxygen pressure difference at maximal exercise and arterial oxygen pressure decline during effort. Overall, exercise capacity seems to be impaired in patients with PEx, and hemoglobin and oxygen‐related parameters remain underreported.

## 1 INTRODUCTION

Pectus excavatum (PEx) is the most common congenital chest wall deformity (Janssen et al., 2024; NHS England, 2019). It is characterized by an anterior depression of the sternum, resulting in a reduced anteroposterior thoracic diameter (Dunning et al., 2024; Janssen et al., 2024). Its incidence is estimated between 1/400 and 1/1000 (Dunning et al., 2024; NHS England, 2019), with a sex ratio between 3:1 and 5:1, predominantly affecting males (Janssen et al., 2024; NHS England, 2019). The primary concern of patients with PEx is often the aesthetic appearance of deformity, though some also report functional limitations (Zuidema et al., 2018). Objective exercise limitation is fairly predicted by subjective discomfort or anthropometric indices (Casar Berazaluce et al., 2020; Hardie et al., 2021).

Exercise capacity is assessed by incremental cardiopulmonary exercise tests (CPET) (American Thoracic Society, American College of Chest Physicians, 2003), and can support clinical decisions in PEx (Dunning et al., 2024). During these tests, parameters reported at maximal exercise include power output (expressed in watts), oxygen uptake (VO_2_max) carbon dioxide production (VCO_2_), heart rate (HR), tidal volume (VT), minute ventilation (VE), breathing frequency (BF), and the respiratory exchange ratio (RER) (Albouaini et al., 2007). Impaired oxygen pulse (O_2_P = VO_2_/HR) evolution during exercise is frequently observed in PEx (Dupuis et al., 2024; Kelly Jr et al., 2016; Oleksak et al., 2022) and attributed to a reduced left ventricular ejection fraction (Chuang et al., 2022; Umapathi & Nguyen, 2023), while it could result from impaired arteriovenous oxygen difference (A‐V O_2_ diff) (Neviere et al., 2011; Oleksak et al., 2022; Tardy et al., 2015). Improved insight into the pathophysiology of exercise impairment could help determine which patients with PEx should undergo rehabilitation versus surgery (Ateş et al., 2025).

The objectives of this study were (1) to provide an overview of current knowledge regarding exercise limitations in patients with non‐operated PEx (i.e., VO_2_max) and (2) to provide an overview of articles assessing hemoglobin and/or oxygen content in blood during exercise testing.

## 2 METHOD

The scoping review design was chosen as the most appropriate approach for synthesizing information from primary source articles and identifying knowledge gaps to guide future research. This scoping review was conducted following the framework proposed by Levac and colleagues (Levac et al., 2010), as well as the Preferred Reporting Items for Systematic Reviews and Meta‐Analyses (PRISMA) guidelines (Tricco et al., 2018), using the extension for scoping reviews (see Data S1). The protocol for this review was registered on the Open Science Framework (OSF) platform (https://doi.org/10.17605/OSF.IO/2N6JY).

### 2.1 Identifying the research question

The main research question of this scoping review was: “What is the impact of PEx on cardiopulmonary fitness in children and adults with non‐operated PEx?”

This research question was divided into the following two sub‐questions: “What is the current knowledge regarding exercise limitations in patients with non‐operated PEx?,” “What is the current knowledge regarding hemoglobin and/or oxygen content in blood during exercise testing in non‐operated patients with PEx?”

### 2.2 Identifying relevant articles

The search strategy was developed collaboratively by three members of the research team (ML, MH, and PA). A combination of keywords and Medical Subject Headings (MeSH) terms was used. The search strategy was conducted using PubMed, ScienceDirect, Cumulative Index for Nursing and Allied Health Literature (CINAHL), and SportDiscus. One research team member (MH) carried out the search strategy across all four databases. The search strategy was first developed for PubMed and then adapted for use in other databases (see Data S2). The search strategy was performed in all databases considering published articles from inception to February 15th, 2025. Reference lists of the relevant articles were also hand‐searched to ensure a comprehensive overview of exercise limitations in patients with non‐operated PEx. All citations retrieved through the search strategy were imported into an EndNote library (Clarivate, London, UK), where duplicates were identified and removed. All remaining citations were then uploaded to Rayyan (Rayyan Systems, Inc., Cambridge, MA, USA) for subsequent screening.

### 2.3 Articles selection

#### 2.3.1 Definition of key concept

This review focuses on articles examining the physiological responses to aerobic exercise in patients with PEx, specifically those assessing functional capacity through any exercise testing method, and hemoglobin and/or oxygen content in blood through any available method. Regarding exercise testing, relevant assessment methods included CPET performed on a cycle ergometer or treadmill, using either an incremental/ramp or constant load protocol. The six‐minute walk test (6MWT) was also considered. The primary variables of interest included VO_2_max and maximal oxygen pulse (O_2_Pmax). Regarding hemoglobin and/or oxygen content in blood, the parameters considered were capillary oxygen saturation (SpO_2_), transcutaneous oxygen pressure (PtcO_2_), arterial oxygen partial pressure (PaO_2_), alveolar‐arterial oxygen gradient (A‐aDO_2_), and hemoglobin (Hb) concentration. Additionally, ventilatory variables such as forced vital capacity (FVC), forced expiratory volume in one second (FEV1), total lung capacity (TLC), and diffusing capacity of the lung for carbon monoxide (DLCO) were included to provide a comprehensive profile of the studied population.

#### 2.3.2 Inclusion and exclusion criteria

To be included, articles should involve human participants and be published in peer‐reviewed scientific journals. Study designs were limited to retrospective and cross‐sectional articles as well as cohorts or randomized controlled trials. In the case of cohorts and randomized controlled trials, only baseline data were considered. In addition, patients' assessments should at least include an exercise testing method reporting VO_2_max values, power, or maximum walking distance. To be included, articles needed to fulfill inclusion criteria regarding the specific targeted population (P), intervention (I), and outcome measures (O) identified as pertinent. Here are the details of the inclusion criteria:

- P: children (individuals <18 years old) and adults (individuals ≥18 years old) with non‐operated PEx
- I: exercise testing (assessed by CPET or any other validated maximal or submaximal exercise testing)
- O: exercise capacity (VO_2_max and O_2_Pmax (absolute or % predicted) for CPET studies, or any other objective measure of exercise capacity for other testing methods); arteriovenous content during exercise (assessed through SpO_2_, PtcO_2_, PaO_2_, A‐aDO_2_, or Hb concentration).

Exclusion criteria include systematic reviews, conference abstracts or proceedings, letters to the editor, monographs, book chapters, research reports, and case reports.

#### 2.3.3 Screening

All potentially eligible articles were independently screened by two researchers (ML and MH). First, a blind screening of titles and abstracts was conducted. Articles were classified as “included” when they clearly and explicitly met the eligibility criteria, they were classified as “excluded” when they clearly and explicitly did not, and they were classified as “maybe” when inclusion could not be determined based on the available information. Every 20 articles, the two reviewers (ML and MH) met to discuss disagreements and resolve any conflicts. If no consensus was reached between the two authors, a third independent reviewer (SL) acted as an arbitrator. Articles initially classified as “Included” or “Maybe” were then reviewed in full text using the same blinded procedure applied during title and abstract screening.

### 2.4 Charting the data

The following descriptive and outcome variables were extracted from all relevant articles using a standardized table: authors, publication year, article title, study country, main objective, study design, population characteristics (age, sex, sample size), outcome measures (e.g., VO_2_max, O_2_Pmax, PaO_2_), assessment techniques (e.g., CPET, blood gas analysis), key findings related to the review objectives, and results informing the studied population or pathophysiological hypotheses for exercise intolerance. Two researchers (ML and MH) independently extracted the data, and a third reviewer (PA) was consulted to resolve any disagreements when necessary.

### 2.5 Collating, summarizing, and reporting data

#### 2.5.1 Study designs and participant characteristics

To provide an overall picture of non‐operated patients with PEx, data on publication years, study designs, the number of participants, and participant characteristics were summarized.

#### 2.5.2 Organizing the results

First, data related to exercise assessment and capacity, including protocols and performance scores (absolute or percentage‐based values such as VO_2_max and O_2_Pmax) were extracted and summarized in a structured table. Second, all other available data concerning parameters involved in the measurement or evolution of hemoglobin and/or oxygen content in blood were extracted following a similar process. Third, data from spirometry were extracted and presented in the summary table as this information help to provide an overview of the functional abilities of patients with PEx.

### 2.6 Critical appraisal

The methodological quality of the included studies was assessed using the Joanna Briggs Institute (JBI) critical appraisal checklists corresponding to each study design (cohort (Moola et al., 2020), cross‐sectional (Barker et al., 2026) and quasi‐experimental (Barker et al., 2024)), with scores calculated as the proportion of criteria rated “yes” over the total number of applicable items. Risk of bias was classified using predefined cut‐offs: ≥80% = low, 60–79% = moderate, <60% = high.

## 3 RESULTS

**FIGURE 1:**
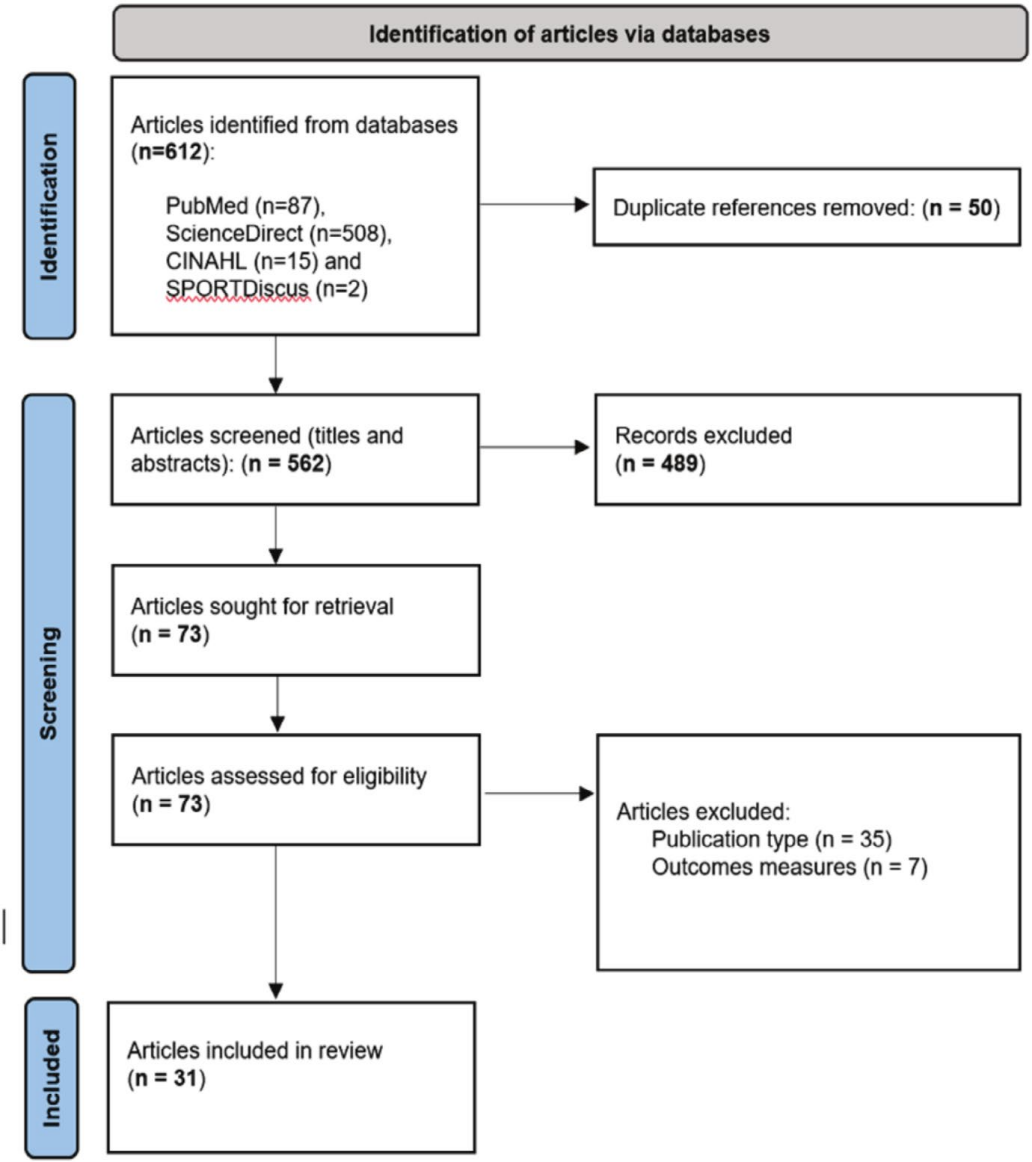
PRISMA flowchart diagram.

A total of 612 articles were identified from the four databases for inclusion in this scoping review. After removing 50 duplicates, 562 articles remained for title and abstract screening. The screening of titles and abstracts excluded 489 articles that failed to meet the criteria, leaving 73 articles for full‐text review. Of these, 31 articles fulfilled the inclusion criteria. No additional articles were identified from the reference lists of the included articles. Figure 1 illustrates the article selection flowchart.

Based on study design, 11 articles were prospective cohort studies (Abu‐Tair et al., 2018; Borowitz et al., 2003; Cahill et al., 1984; Das et al., 2019; Dupuis et al., 2024; Eldredge et al., 2025; Kelly Jr et al., 2013; Morshuis et al., 1994; Neviere et al., 2011; O'Keefe et al., 2013; Sigalet et al., 2007), seven were cross‐sectional studies (Bevegård, 1962; Castile et al., 1982; Cavestri et al., 2010; Ghory et al., 1989; Malek et al., 2003; Oleksak et al., 2022; Zens et al., 2022), five were comparative studies (Al‐Assiri et al., 2009; Haller & Loughlin, 2000; Quigley et al., 1996; Tang et al., 2012; Zhao et al., 2000), five were retrospective studies (Jaroszewski et al., 2022; Ravanbakhsh et al., 2022; Sanjurjo et al., 2024; Satur et al., 2021; Swanson et al., 2012), and three were prospective controlled cohort studies (Maagaard et al., 2013; Udholm et al., 2016; Wynn et al., 1990). All included articles were published between 1962 and 2025 from 11 different countries.

The 31 articles included a total of 2937 patients with non‐operated PEx among whom 860 were clearly identified as children in 13 articles (Borowitz et al., 2003; Cahill et al., 1984; Das et al., 2019; Eldredge et al., 2025; Ghory et al., 1989; Haller & Loughlin, 2000; Kelly Jr et al., 2013; Maagaard et al., 2013; O'Keefe et al., 2013; Sigalet et al., 2007; Swanson et al., 2012; Tang et al., 2012; Wynn et al., 1990), 1238 were clearly identified as adults in four articles (Jaroszewski et al., 2022; Neviere et al., 2011; Ravanbakhsh et al., 2022; Udholm et al., 2016), and the remaining 839 participants were not classified, as 14 articles included both children and adults within the same study population (Abu‐Tair et al., 2018; Al‐Assiri et al., 2009; Bevegård, 1962; Castile et al., 1982; Cavestri et al., 2010; Malek et al., 2003; Morshuis et al., 1994; Oleksak et al., 2022; Quigley et al., 1996; Sanjurjo et al., 2024; Satur et al., 2021; Umapathi & Nguyen, 2023; Zens et al., 2022; Zhao et al., 2000). Table 1 presents a summary of the extracted study information and critical appraisal results. The complete data extraction table is available in Data S3, and the full critical appraisal analysis is provided in Data S4.

**TABLE 1:** Extracted study information and critical appraisal results.

Study information					Critical appraisal
Authors and year	Country	Design	Population		Risk categorization
			Number of participants	Mean age	
Abu‐Tair et al. (2018)	Germany	Prospective cohort study	99 (82 males and 17 females)	Males: 18.8 yo (SD 7.7) Females: 16.1 yo (SD 4.7)	Moderate
Al‐Assiri et al. (2009)	Canada	Comparative study	30 (15 males and 15 females)	SN group: 13.5 yo (SD 1.9) MN group: 15.0 yo (SD 4.1)	Moderate
Bevegård (1962)	Sweden	Cross‐sectional study	16 (10 men and 6 women)	Range = 15–63 yo	High
Borowitz et al. (2003)	USA	Prospective cohort study	10 (all males)	13.4 yo (SD 3)	High
Cahill et al. (1984)	USA	Prospective cohort study	14 PEx (and 5 pectus carinatum) (no information about sex)	10.9 yo (range = 6–17 yo)	Moderate
Castile et al. (1982)	USA	Cross‐sectional study	8 (7 with PEx and 1 with pectus carinatum)	Range = 9–22 yo	High
Cavestri et al. (2010)	France	Cross‐sectional study	32 (25 males and 7 females)	25.9 yo (SD 11.8)	Low
Das et al. (2019)	USA	Prospective cohort study	24 (no information about sex)	12.9 yo (SD 3.6)	High
Dupuis et al. (2024)	France	Prospective study	60 (40 males et 20 females)	28 yo (SD 11.3)	High
Eldredge et al. (2025)	USA	Prospective cohort observational study	81 (72% males and 28% females)	15.28 yo (SD 1.5)	Moderate
Ghory et al. (1989)	USA	Cross‐sectional study	28 (14 PEx and 14 controls) (no information about sex)	PEx group: 10.2 yo (SD 4.2)	Low
Haller and Loughlin (2000)	USA	Comparative study	36 (no information about sex)	16 yo (SD 3)	Low
Jaroszewski et al. (2022)	USA	Retrospective cohort study	392 (267 males and 125 females)	31.0 yo (SD 9.8)	Moderate
Kelly Jr et al. (2013)	USA	Multicenter prospective cohort study	327 (no information about sex)	Patients who completed the whole study: 13.60 yo (SD 3.26) Patients lost: 14.37 yo (SD 3.50)	Moderate
Maagaard et al. (2013)	Danmark	Single center prospective controlled study	49 PEx (39 males and 10 females) 26 control (16 males and 10 females)	PEx group: 15.5 yo (SD 1.7) Control group: 15.0 yo (SD 1.9)	Low
Malek et al. (2003)	USA	Cross‐sectional study	21 (15 males and 6 females)	23.6 yo (SD 8.9)	Low
Morshuis et al. (1994)	Netherlands	Prospective cohort study	35 (28 males and 7 females)	17.9 yo (SD 5.6)	High
Neviere et al. (2011)	France	Prospective cohort study	70 (61 males and 9 females)	Operated patients: 27.11 yo (range = 18–62)	Moderate
O'Keefe et al. (2013)	Canada	Prospective cohort study	130 (67 who only completed the whole follow up, and 63 lost) (no information about sex)	Completed the whole follow up: patients: 13.9 yo (SD 2.3) Lost participants: 13 yo (SD 3.4)	Moderate
Oleksak et al. (2022)	Slovakia	Cross‐sectional study	32 (28 males and 4 females)	15.3 yo (SD 2.9)	Low
Quigley et al. (1996)	USA	Comparative study	36 (32 males and 4 females)	16 yo (SD 3)	Moderate
Ravanbakhsh et al. (2022)	USA	Retrospective study	757 (527 males and 230 females)	Females: 35.0 yo (SD 11.8) Males: 32.4 yo (SD 10.2) ***Mean ages are provided for the whole studied population (PEx as well as carinatum and arcuatum) among a total of 776 patients enrolled	Low
Sanjurjo et al. (2024)	Argentina	Retrospective study	43 (38 males and 5 females)	17.8 yo (SD 6.7), range = 11–36 yo	Moderate
Satur et al. (2021)	UK	Retrospective study	70 (60 males and 10 females)	28 yo (SD 6.6)	Low
Sigalet et al. (2007)	Canada	Prospective cohort study	26 (no information about sex)	13.2 yo (SD 2.1)	Moderate
Swanson et al. (2012)	USA	Retrospective study	87 (80% males and 20% females)	15.2 yo (SD 0.3)	Low
Tang et al. (2012)	Danmark	Comparative study	PEx group = 49 Control group = 26	PEx group: 15.5 yo (SD 1.7) Control group: 15.0 yo (SD 1.9)	Moderate
Udholm et al. (2016)	Danmark	Single center prospective controlled study	19 participants (13 males and 6 females)	32 yo	Moderate
Wynn et al. (1990)	USA	Prospective controlled study	13 (11 males and 2 females | 9 operated and 4 non‐operated)	Operated group: 13.8 yo Non‐operated group: 14 yo (age range for all participants = 10–16 yo)	Low
Zens et al. (2022)	USA	Cross‐sectional study	345 (81% males and 19% females)	15.2 yo (SD 3.95)	Low
Zhao et al. (2000)	Israel	Comparative study	PEx group: 13 (11 males and 2 females) Control group: 20 (12 males and 8 females)	PEx group: 19 yo (SD 6) Control group: 25 yo (SD 11)	Moderate

Abbreviations: PEx, pectus excavatum; SD, standard deviation; yo, years old.

### 3.1 Exercise capacity

Table 2 presents a summary of the extracted data related to exercise capacity.

**TABLE 2:** Extracted data from included articles related to exercise capacity.

Included article	Exercise capacity			
Authors and year	Measurement technique	VO_2_max normal limit	VO_2_max (% of predicted value)	VO_2_max (absolute value)
Abu‐Tair et al. (2018)	CPET on treadmill	≥ 85% of predicted value	Males: 93.3% (SD 14.8), Females: 101.0% (SD 18.3)	Males: 43.3 mL/kg/min (SD 6.6) Females: 36.6 mL/kg/min (SD 6.0)
Al‐Assiri et al. (2009)	CPET on treadmill and cycloergometer	NA	SN: 68.40% (SD 9.99) MN: 72.00% (SD 19.75)	SN: 32.81 L/kg·min (SD 6.65) MN: 34.51 L/kg·min (SD 6.97)
Bevegård (1962)	CPET on cycloergometer	NA	NA	NA
Borowitz et al. (2003)	CPET on cycloergometer	NA	85% (range = 52–106)	1882 mL/min (range = 1011–2621)
Cahill et al. (1984)	CPET on cycloergometer	NA	NA	1.26 mL/kg/min (SD 0.44)
Castile et al. (1982)	CPET on cycloergometer (Steady state)	NA	NA	range = 0.79–2.32 L/min
Cavestri et al. (2010)	CPET on cycloergometer	≥84% of predicted value	78.6% (SD 22.1)	33.8 mL/kg/min (SD 10.4)
Das et al. (2019)	CPET on treadmill	NA	62% (SD 25)	32 mL/kg/min (SD 13)
Dupuis et al. (2024)	CPET on cycloergometer	>84% of predicted value	87% (SD 13)	33.4 mL/min/kg (SD 6.1)
Eldredge et al. (2025)	CPET on treadmill	>80% of predicted value	75.9% (SD 16.9)	35.0 mL/kg/min (SD 7.4)
Ghory et al. (1989)	CPET on cycloergometer	NA	91% (SD 12)	1.749 mm/min (SD 854)
Haller and Loughlin (2000)	CPET on treadmill	NA	NA	40 mL/kg/min (SD 7)
Jaroszewski et al. (2022)	CPET on cycloergometer	≥80 of predicted value	73.6% (SD 15.8)	26.5 mL/kg/min (SD 6.2)
Kelly Jr et al. (2013)	CPET on both cycloergometer or treadmill depending on the center	NA	NA	3.18 L/min (SD 3.33)
Maagaard et al. (2013)	CPET on cycloergometer	NA	NA	26 ml/min/kg (SD 7,1)
Malek et al. (2003)	CPET on cycloergometer	NA	75% (SD 19)	2.14 L/min (SD 0.75)
Morshuis et al. (1994)	CPET on cycloergometer	NA	NA	26.8 mL/min/kg (SD 6.1)
Neviere et al. (2011)	CPET on cycloergometer	NA	77% (SD 2)	34.9 mL/kg/min (SD 7.5)
O'Keefe et al. (2013)	CPET on cycloergometer or treadmill	NA	Completed the whole follow up: 70.1% (SD 15.0) Lost patients: 75.8% (SD 18.2)	NA
Oleksak et al. (2022)	CPET on treadmill	NA	VO_2_max in z‐score = −1.51 (SD 1.11)	NA
Quigley et al. (1996)	CPET on treadmill	NA	NA	40 mL/kg/min (SD 7)
Ravanbakhsh et al. (2022)	CPET (no more details were provided)	NA	Females: 77% (SD 16.9) Males: 72.2% (SD 15.7)	NA
Sanjurjo et al. (2024)	6MWT	NA	NA	NA
Satur et al. (2021)	CPET on cycloergometer	>80% of predicted value	78.0% (SD 13.7)	NA
Sigalet et al. (2007)	CPET on cycloergometer or treadmill	NA	70.8% (SD 11)	34.1 mL/kg/min (SD 6.1)
Swanson et al. (2012)	CPET on cycloergometer	>85% or predicted value	86.3% (se 1.7)	NA
Tang et al. (2012)	CPET on cycloergometer	NA	NA	26 mL/kg/min (SD 6.0)
Udholm et al. (2016)	CPET on cycloergometer	NA	NA	30.4 mL/kg/min (SD 6)
Wynn et al. (1990)	CPET on cycloergometer	NA	NA	Operated group: 36.1 mL/kg/min (SD 4.4) Non‐operated group: 41.2 mL/kg/min (SD 7.3)
Zens et al. (2022)	CPET on cycloergometer	>80% of predicted value	89.1% (SD 16.5)	NA
Zhao et al. (2000)	CPET on cycloergometer	NA	NA	In sitting position: 1480 mL/min (SD 462) In supine position: 1351 mL/min (SD 345)

Abbreviations: CPET, cardiopulmonary exercise tests; MN, modified Nuss procedure; NA, not applicable; SD, standard deviation; SN, standard Nuss procedure; VO_2_max, oxygen uptake at maximal incremental exercise.

#### 3.1.1 Exercise procedure

Among the 31 included articles, 27 used incremental protocols (either step by step or linear), one reported varying protocols depending on the investigation center without providing specific details (Kelly Jr et al., 2013), one used the 6MWT (Sanjurjo et al., 2024) and two did not describe their procedure (Ravanbakhsh et al., 2022; Swanson et al., 2012).

#### 3.1.2 CPET results

Seventeen out of the 31 included articles reported CPET results, expressed VO_2_max as a percentage of predicted values, ranging from 62% (±25) to 101.0% (±18.3) (Abu‐Tair et al., 2018; Al‐Assiri et al., 2009; Borowitz et al., 2003; Cavestri et al., 2010; Das et al., 2019; Dupuis et al., 2024; Eldredge et al., 2025; Ghory et al., 1989; Jaroszewski et al., 2022; Malek et al., 2003; Neviere et al., 2011; O'Keefe et al., 2013; Ravanbakhsh et al., 2022; Satur et al., 2021; Sigalet et al., 2007; Swanson et al., 2012; Zens et al., 2022). Of these 17 articles, two reported using an 85% cut‐off of the VO_2_max to define impaired exercise capacity (Abu‐Tair et al., 2018; Swanson et al., 2012). Based on this cut‐off, the average VO_2_max in these two articles was within normal limits. In two other articles, the cut‐off value was set at 84% of the predicted VO_2_max (Cavestri et al., 2010; Dupuis et al., 2024), with one reporting average values below this threshold (Cavestri et al., 2010). Four other articles reported using a cut‐off of 80% of the predicted VO_2_max (Eldredge et al., 2025; Jaroszewski et al., 2022; Satur et al., 2021; Zens et al., 2022) among which three reported impaired exercise capacity on average when applying this threshold (Eldredge et al., 2025; Jaroszewski et al., 2022; Satur et al., 2021). In the remaining nine articles (Al‐Assiri et al., 2009; Borowitz et al., 2003; Das et al., 2019; Ghory et al., 1989; Malek et al., 2003; Neviere et al., 2011; O'Keefe et al., 2013; Ravanbakhsh et al., 2022; Sigalet et al., 2007), no cut‐off value was specified. Fourteen articles did not report exercise capacity as a percentage of predicted VO_2_max, but instead presented results in absolute units such as mL/kg/min or mL/min/kg (Cahill et al., 1984; Haller & Loughlin, 2000; Maagaard et al., 2013; Quigley et al., 1996; Tang et al., 2012; Udholm et al., 2016; Wynn et al., 1990). Reported values ranged from a minimum of 26 mL/kg/min (±7.1) (Maagaard et al., 2013) to a maximum of 40 mL/kg/min (±7) (Quigley et al., 1996). In terms of mL/min or L/min, the range extended from 0.79 L/min to a maximum of 3.18 L/min (±3.33) (Kelly Jr et al., 2013). It is to note that one article (Cahill et al., 1984) reported a mean VO_2_max of 1.26 mL/kg/min (±0.44) which is not a physiological result. Another article (Oleksak et al., 2022) reported VO_2_max values using Z‐score, with a mean of −1.51 (±1.11). One article (Bevegård, 1962) did not report VO_2_max values but instead presented power output in sitting position, ranging from 220 kpm/min to 1110 kpm/min, and in supine position, ranging from 380 kpm/min to 1090 kpm/min, while another article reported maximum walking distance as the outcome measure (Sanjurjo et al., 2024) based on the 6MWT assessment, with a mean distance of 600.8 ± 67.6 m.

### 3.2 Hemoglobin and/or oxygen content in blood during exercise

Among the 31 included articles, 17 (54.8%) articles mentioned assessing hemoglobin and/or oxygen content in blood, either through SpO_2_ measurement or arterial blood gas sampling (Bevegård, 1962; Castile et al., 1982; Cavestri et al., 2010; Das et al., 2019; Haller & Loughlin, 2000; Jaroszewski et al., 2022; Maagaard et al., 2013; Malek et al., 2003; Morshuis et al., 1994; O'Keefe et al., 2013; Quigley et al., 1996; Sanjurjo et al., 2024; Satur et al., 2021; Sigalet et al., 2007; Swanson et al., 2012; Tang et al., 2012; Wynn et al., 1990). Data extraction for hemoglobin and/or oxygen content in blood during exercise is presented in Table 3.

**TABLE 3:** Data extraction for hemoglobin and/or oxygen content in blood during exercise.

Article	Hemoglobin and/or oxygen content	
	Measurement technique	Key results
Abu‐Tair et al. (2018)	NA	NA
Al‐Assiri et al. (2009)	NA	NA
Bevegård (1962)	Hb: yes SpO_2_: yes Blood gas analysis: NA	Hb concentration: range from 11.6 to 17.3 g/100 mL SpO_2_: from 93% to 99%
Borowitz et al. (2003)	NA	NA
Cahill et al. (1984)	NA	NA
Castile et al. (1982)	Hb: NA SpO_2_: NA Blood gas analysis: yes	Blood gas analysis: Normal pattern during exercise (no objective values were reported by the authors)
Cavestri et al. (2010)	Hb: NA SpO_2_: yes Blood gas analysis: yes	No objective data about either SpO_2_or blood gas analysis were reported. A‐aDO_2_max: 25.0 mmHg (SD 14.7)
Das et al. (2019)	Hb: NA SpO_2_: yes Blood gas analysis: yes	No objective data about either SpO_2_ or blood gas analysis were reported.
Dupuis et al. (2024)	NA	NA
Eldredge et al. (2025)	NA	NA
Ghory et al. (1989)	NA	NA
Haller and Loughlin (2000)	Hb: NA SpO_2_: yes Blood gas analysis: NA	No objective data reported for SpO_2_
Jaroszewski et al. (2022)	Hb: NA SpO_2_: yes Blood gas analysis: NA	No objective data reported for SpO_2_
Kelly Jr et al. (2013)	NA	NA
Maagaard et al. (2013)	Hb: NA SpO_2_: yes Blood gas analysis: NA	No objective data reported for SpO_2_
Malek et al. (2003)	Hb: NA SpO_2_: yes Blood gas analysis: NA	SpO_2_: 95.5% (SD 2.1)
Morshuis et al. (1994)	Hb: NA SpO_2_: yes Blood gas analysis: yes	SpO_2_: remains normal during exercise even at peak exercise. (No objective data reported) PaO_2_ (between rest and exercise): −1.0 (SD 1.6)
Neviere et al. (2011)	NA	NA
O'Keefe et al. (2013)	Hb: NA SpO_2_: yes Blood gas analysis: NA	No objective data reported for SpO_2_
Oleksak et al. (2022)	NA	NA
Quigley et al. (1996)	Hb: NA SpO_2_: yes Blood gas analysis: NA	Resting SpO_2_: 97% (SD 1) Minimal SaO_2_: 96% (SD 2)
Ravanbakhsh et al. (2022)	NA	NA
Sanjurjo et al. (2024)	Hb: NA SpO_2_: yes Blood gas analysis: NA	Baseline oxygen: 97.4% (SD 1.2) Final oxygen: 96.4% (SD 2.1)
Satur et al. (2021)	Hb: NA SpO_2_: yes Blood gas analysis: NA	No objective data reported for SpO_2_
Sigalet et al. (2007)	Hb: NA SpO_2_: yes Blood gas analysis: NA	No objective data were reported for SpO_2_
Swanson et al. (2012)	Hb: NA SpO_2_: yes Blood gas analysis: NA	No objective data were reported for SpO_2_
Tang et al. (2012)	Hb: NA SpO_2_: yes Blood gas analysis: NA	No objective data were reported for SpO_2_
Udholm et al. (2016)	NA	NA
Wynn et al. (1990)	Hb: NA SpO_2_: yes Blood gas analysis: NA	No saturation decrease during exercise. (no objective data were reported)
Zens et al. (2022)	NA	NA
Zhao et al. (2000)	NA	NA

Abbreviations: A‐aDO_2_max, alveolar arterial oxygen difference at maximal exercise; A‐V O_2_ diff, arteriovenous oxygen difference; DLCO, diffusing capacity of the lung for carbon monoxide; DLCO/A, diffusing capacity of the lung for carbon monoxide/alveolar ventilation; Hb, hemoglobin; NA, not applicable; PFO, patent foramen ovale; SaO_2_, arterial oxygen saturation; SpO_2_, saturation.

Regarding hemoglobin, one article (3.2%) reported assessing hemoglobin concentration prior to exercise testing, with levels ranging from 11.6 to 17.3 g/100 mL (Bevegård, 1962).

Regarding SpO_2_, 15 articles (48.4%) reported assessing it in their methods section (Cavestri et al., 2010; Das et al., 2019; Haller & Loughlin, 2000; Jaroszewski et al., 2022; Maagaard et al., 2013; Malek et al., 2003; Morshuis et al., 1994; O'Keefe et al., 2013; Quigley et al., 1996; Sanjurjo et al., 2024; Satur et al., 2021; Sigalet et al., 2007; Swanson et al., 2012; Tang et al., 2012; Wynn et al., 1990) with only four articles reporting objective data (Bevegård, 1962; Malek et al., 2003; Quigley et al., 1996; Sanjurjo et al., 2024). In fact, three articles reported mean SpO_2_ values ranging from 95.5% (±2.1) (Malek et al., 2003) to 97.4% (±1.2) (Sanjurjo et al., 2024), whereas one article provided the range of individual values observed among participants with PEx, which extended from 93% to 99% (Bevegård, 1962). The timing of SpO_2_ assessment varied across the included articles. In fact, SpO_2_ was continuously monitored during exercise (Malek et al., 2003), was measured at rest (Quigley et al., 1996), was assessed “just before and after” the exercise capacity assessment (Sanjurjo et al., 2024) or the assessment was not specified (Bevegård, 1962).

Regarding blood gas analysis, five articles (16.1%) reported having assessed blood gas analysis (Bevegård, 1962; Castile et al., 1982; Cavestri et al., 2010; Das et al., 2019; Morshuis et al., 1994). Only two of them reported objective results in their results section (Cavestri et al., 2010; Morshuis et al., 1994). Cavestri et al. reported a mean A‐aDO_2_ at maximal exercise of 25.0 mmHg (±14.7) (Cavestri et al., 2010), while Morshuis et al. reported a minor decrease in PaO_2_ of −1.0 mmHg (±1.6) (Morshuis et al., 1994). No other information about blood gas analysis was provided by the authors. Cavestri et al. also reported that five of their 32 participants exhibited an A‐aDO_2_ at peak exercise higher than 35 mmHg (Cavestri et al., 2010).

## 4 DISCUSSION

This scoping review explored the current state of knowledge regarding exercise capacity, as well as hemoglobin and/or oxygen content in blood measurement during exercise testing. The results of the present scoping review showed that while some patients with PEx exhibited exercise limitations, others did not. Moreover, the results showed that hemoglobin concentration was almost never reported when assessing exercise capacity in patients with PEx, and that oxygen content, especially SpO_2_ and PaO_2_, was also scarcely reported in this context.

### 4.1 Exercise capacity

The present scoping review highlighted globally heterogeneous VO_2_max scores in patients with PEx both across and within the included articles. Such heterogeneity was also reported in a recent systematic review with meta‐analysis conducted by Media et al. (2025). This suggests that an important proportion of patients with PEx included in the selected articles had no exercise limitation, whereas another substantial subset did. When considering VO_2_max values expressed as a percentage of the predicted value, the most recent guidelines for evaluating patients with PEx recommend using an 85% threshold to determine whether VO_2_max is impaired (Dunning et al., 2024). Applying this cut‐off, 64.7% (11/17) of the studies reported mean VO_2_max below 85% of predicted (Al‐Assiri et al., 2009; Cavestri et al., 2010; Das et al., 2019; Eldredge et al., 2025; Jaroszewski et al., 2022; Malek et al., 2003; Neviere et al., 2011; O'Keefe et al., 2013; Ravanbakhsh et al., 2022; Satur et al., 2021; Sigalet et al., 2007), while four reported VO_2_max values between 85% and 90% of the predicted value (Borowitz et al., 2003; Dupuis et al., 2024; Swanson et al., 2012; Zens et al., 2022) and two reported values above 90% (Abu‐Tair et al., 2018; Ghory et al., 1989). Assuming a normal distribution of these values, approximately one‐sixth of individual measurements may fall below the mean minus one standard deviation. Consequently, articles that do not explicitly report exercise limitations within their populations may still include at least one‐sixth of participants with VO_2_max values below the 85% threshold. This proportion may be even higher when the mean VO_2_max value is close to the threshold, as observed in the articles by Borowitz et al. (85%), Dupuis et al. (87%), and Swanson et al. (86%). Even when applying a more lenient threshold of 80%, this observation remains valid. However, it could be argued that the current 85% threshold may not necessarily apply to the interpretation of results from articles published prior to the latest recommendations by Dunning and colleagues (Dunning et al., 2024).

Besides, exercise capacity data presented as absolute VO_2_max values (e.g., in mL/kg/min or L/min) are difficult to interpret, as they must be contextualized with individual characteristics such as age, sex, and body weight (American Thoracic Society, American College of Chest Physicians, 2003). Without reference values, absolute VO_2_max scores cannot be meaningfully assessed or classified as indicative of impaired exercise capacity. Nevertheless, we hypothesize that similar heterogeneity may be present in those articles as well. As a comparison, a study focusing on patients with moderate scoliosis reported a nearly twofold higher standard deviation in VO_2_max scores (SD = 9.12 mL/kg/min) compared to the healthy control group (SD = 4.9 mL/kg/min) (Kesten et al., 1991).

The observed heterogeneity of VO_2_max values and standard deviation across included articles can be due to several factors concerning composition of studied population within studies. First, treadmill‐based CPET may overestimate VO_2_max values by up to 5%–10% compared to other CPET methods (Media et al., 2025; Pearson et al., 2023), and outcome differences exists depending on brand and devices (Van Hooren et al., 2023). Second, differences may exist depending on age and sex, as in the healthy population (Sven et al., 2010). Third, a correlation between exercise capacity and severity of deformity, even though a controversy exists in the literature (Oleksak et al., 2022; Swanson et al., 2012; Zens et al., 2022). Last, cardiac comorbidities may exist in some patients with PEx, since mitral valve prolapse prevalence is likely six times higher in patients with PEx than in the healthy population (Sonaglioni et al., 2024). Further studies with stricter eligibility criteria and appropriate statistical analyses are needed to identify potential pathophysiological causes and predictors of exercise impairment in patients with PEx.

### 4.2 Hemoglobin and/or oxygen content in blood

This sub‐section of our scoping review highlights the lack of objectively reported measures of hemoglobin concentration and/or oxygen content in blood during exercise testing in patients with PEx. The potential impact of hemoglobin levels on exercise capacity in PEx remains poorly documented. While one study reported values as low as 11.6 g/dL (Bevegård, 1962), current data are insufficient to determine if anemia contributes to the observed exercise impairment. Given the established role of hemoglobin in oxygen transport and VO_2_max (Webb et al., 2023), further studies assessing hematological profiles before and during CPET are warranted to clarify this potential relationship in PEx patients, especially considering that changes have been reported in Early‐Onset Scoliosis before and after surgery (Caubet et al., 2009; Glotzbecker et al., 2019). Another gap highlighted by this scoping review is the assessment of blood oxygen content, particularly SpO_2_. Indeed, only 15 out of 31 articles (48.4%) reported measuring SpO_2_ in their methods section, and only four (12.9%) reported quantitative results (Bevegård, 1962; Malek et al., 2003; Quigley et al., 1996; Sanjurjo et al., 2024). Some methodological biases prevent accurate interpretation of the reported results. For example, three articles reported only a single value of SpO_2_ without specifying whether it represented the minimum, maximum, or mean SpO_2_ value measured during exercise testing (Bevegård, 1962; Malek et al., 2003; Quigley et al., 1996). In addition, Sanjurjo and colleagues referred to transcutaneous oximetry testing but reported only saturation values expressed as percentages (Sanjurjo et al., 2024). Lastly, Quigley et al. reported measuring peripheral capillary oxygen saturation (SpO_2_) in their methods section but presented both SpO_2_ and arterial oxygen saturation (SaO_2_) values (Quigley et al., 1996). For SaO_2_, blood gas analysis is required, yet Quigley et al. did not report such an assessment in their methods section. To our knowledge, reliable data on patients with PEx are currently lacking in the exercise‐focused literature. Important consideration is that the absence of reported SpO_2_ data should not necessarily be interpreted as an absence of measurement. In many studies, pulse oximetry was likely monitored routinely as part of standard clinical practice to ensure patient safety, but SpO_2_ was not reported because it was neither a primary nor a secondary outcome. This selective reporting is understandable from a methodological perspective, as studies must balance the number of reported endpoints against statistical and practical constraints, but it nevertheless limits the ability to synthesize evidence regarding perioperative oxygenation. Setting aside this notable limitation, an intriguing heterogeneity emerges across the four articles that report quantitative saturation values. Based on mean values and their standard deviations, they all suggest that a proportion of their participants experienced saturation levels below 95%. This threshold is considered clinically relevant at rest, as it may predict exercise‐induced hypoxemia in patients with chronic obstructive pulmonary disease (Vold et al., 2015). Further studies incorporating reliable and continuous saturation monitoring during physical effort are necessary to detect potential exercise‐induced desaturation in patients with PEx. However, this proportion of low SpO_2_ might not directly be related to PEx but to measurement artifacts, body posture during the test or even comorbid conditions of included participants. These likely rare phenomena might explain why proportion of abnormal SpO_2_ seems low. Conversely, we hypothesize that the exercise limitations observed in a substantial subset of these patients may be associated with impaired oxygen delivery, potentially driven by multiple pathophysiological mechanisms discussed in Data S5. Given that these results cannot lead to any affirmation, further studies are needed to explore this hypothesis.

Regarding blood gas analysis, five out of the 31 included articles (16.1%) reported measuring it in their methods section, but only two out of these 31 articles (6.4%) reported quantitative data in their results (Cavestri et al., 2010; Morshuis et al., 1994). Moreover, like saturation assessment, a notable inconsistency exists among the articles included in our scoping review regarding reported blood gas analysis values. For instance, Cavestri et al. (Cavestri et al., 2010) did not report PaO_2_ values but instead presented the A–aDO_2_ at maximal exercise. Morshuis et al. reported a change in PaO_2_ between rest and exercise but did not specify the timing of the measurements during exercise (e.g., at onset, at VO_2_max, or at the end of the test) (Morshuis et al., 1994). However, like with saturation and exercise testing, considerable heterogeneity exists among the articles included in this scoping review regarding the reported outcome measures. Cavestri et al. reported that five of their 32 included participants presented an abnormal A–aDO_2_ at peak exercise (greater than 35 mmHg) (Cavestri et al., 2010). In the general population, a considered normal A‐aDO_2_ at peak exercise is indeed between 20 and 25 mmHg (Dempsey et al., 1984; Eldridge et al., 2004; Hopkins et al., 2000; Losa‐Reyna et al., 2015; Stickland et al., 2013). This lack of data regarding PaO_2_ during exercise may be due to technical difficulties in setting up blood gas analysis during cardiopulmonary exercise testing (Habedank et al., 2022; Vignati & Cattadori, 2017), which might be overcome by alternative non‐invasive methods such as transcutaneous oxygen pressure (Carter & Banham, 2000; Nikki et al., 1982). As a comparison, hypoxemia has been described in patients with kyphoscoliosis during the 6MWT (Menon & Aggarwal, 2007). Given the anatomical similarity between PEx and kyphoscoliosis, such hypoxemia may also be observable in patients with PEx. Overall, the lack of data on hemoglobin and/or blood oxygen content during exercise hinders a reliable assessment of the true prevalence and severity of exercise‐induced impairments in these parameters and consequently limits our ability to determine their potential role in exercise intolerance among these patients.

### 4.3 Limitations

This scoping review is, to our knowledge, the first study providing an exhaustive overview of exercise capacity in non‐operated patients with PEx, as well as highlighting a substantial lack of knowledge regarding hemoglobin and/or oxygen content in blood during exercise in this population. However, our study is not without limitations. First, the present scoping review included only articles written in French and English. Consequently, some relevant articles published in other languages may have been excluded. The generalizability of the results is limited by the lack of subgroup analyses, due to heterogeneity among the included studies (e.g., age, sex). Second, critical appraisal of included studies showed an important heterogeneity in risk of bias, and only about a third of included studies exhibiting a low risk of bias. Finally, thresholds and calculation methods used to define pathological versus physiological spirometry outcomes have evolved substantially over time (Arce et al., 2025). It is likely that thresholds for VO_2_max, blood oxygen saturation, and related parameters have also changed. Since the articles cover a period of over 60 years, shifts in diagnostic definitions and reference values introduce temporal inconsistencies that limit the comparability of findings across decades.

### 4.4 Clinical implications

Even though this scoping review cannot conclude on whether oxygen change during exercise, and/or hemoglobin are impaired or not in patients with PEx, it highlights the interest for regular assessment in both clinical routine and research. This may allow a better understanding of these mechanisms, to make pathophysiological aspects of PEx clearer. This review highlights the need for individualized clinical evaluation and challenges the view of PEx as a purely cosmetic condition. While conservative management mainly addresses vacuum bell techniques (AlShammari et al., 2026; Khalifa et al., 2025) and posture‐based exercises (Li et al., 2025), aerobic training has been scarcely studied (Amăricăi et al., 2019) despite its likely importance, as suggested through exercise impairment highlighted in this scoping review, and should be considered both clinically and for future studies.

## 5 CONCLUSION

This scoping review highlights that probably many patients with PEx show reduced exercise capacity, although exercise impairment is highly heterogeneous in this population. Moreover, there is a significant lack of studies focusing on hemoglobin and/or oxygen content in blood changes during exercise in this population.

## AUTHOR CONTRIBUTIONS

**Maxime Lokietek:** Data curation; formal analysis; methodology; visualization. **Simon Lecoq:** Conceptualization; methodology; validation. **Laura Filaire:** Conceptualization; formal analysis; resources; supervision; validation; visualization. **Bénédicte Noury‐Desvaux:** Methodology; resources. **Mariève Houle:** Conceptualization; data curation; formal analysis; methodology; project administration; resources; supervision; validation; visualization. **Pierre Abraham:** Conceptualization; formal analysis; methodology; supervision; validation; visualization.

## FUNDING INFORMATION

MH received a postdoctoral scholarship from the Canadian Institutes of Health Research (CIHR) (Funding reference number: 194060) and from the Fonds de recherche du Québec—Santé (FRQS) (https://doi.org/10.69777/343976).

## CONFLICT OF INTEREST STATEMENT

All authors declare that they have no competing interests.

## ETHICS STATEMENT

The authors have nothing to report.

## PERMISSION TO REPRODUCE MATERIALS FROM OTHER SOURCES

All materials used in the preparation of this manuscript were published under at least a CC BY license, permitting their use provided appropriate attribution is given.

## Supporting information


Data S1.

Data S2.

Data S3.

Data S4.

Data S5.

**Data S6.** PRISMA Checklist.

## Data Availability

This scoping review does not contain new data. All relevant data are presented and referenced within the review.
