# Supplementary material for: Exercise impairment in patients with pectus excavatum? A scoping review of evidence and role of arterial content change during effort
Source: Physiol Rep. 2026 Jul 6;14(13):e71005. doi: 10.14814/phy2.71005 (PMC13338107; doi:10.14814/phy2.71005)
Supplement: Supplementary file 1 — Data S1. [file PHY2-14-e71005-s005.pdf]

## SUPPLEMENTARY MATERIAL 1

## RESEARCH STRATEGY

### RESEARCH QUESTIONS:

1. **Main research question**

   What is the impact of PEx on cardiopulmonary fitness in children and adults with non-operated PEx?

2. **Secondary research questions**

- What is the current knowledge regarding exercise limitations in patients with non-operated PEx?
- What is the current knowledge regarding hemoglobin and/or oxygen content in blood during exercise testing in non-operated patients with PEx?
- What pathophysiological hypotheses have been proposed by authors to explain exercise impairment in patients with non-operated PEx?

### OBJECTIVES OF THE SCOPING REVIEW:

1. **Primary objective:**

To provide a comprehensive and reliable overview of current knowledge regarding exercise limitations in patients with non-operated PEx (i.e., VO2max).

2. **Secondary objective**

- To provide an overview of the selected articles assessing hemoglobin and/or oxygen content in blood during exercise testing.
- to summarize the various physiological hypotheses proposed by authors in the selected articles to explain exercise intolerance, if present, in patients with PEx, and to propose a potentially new hypothesis to be considered in future articles.

**PICO definition:**

| | |
|---|---|
| **P** | Children (individuals < 18 years old) and adults (individuals ≥ 18 years old) with non-operated PEx |
| **I** | Exercise testing (assessed by CPET or any other validated maximal or submaximal exercise testing) |
| **C** | NA |
| **O** | Exercise capacity (reported as VO2max and O2Pmax values in absolute terms or as a percentage of predicted value for articles involving CPET, or any other relevant value that can objectively assess exercise capacity for exercise testing methods); arteriovenous content during exercise (assessed through SpO2, PtcO2, PaO2, A-aDO2, or Hb concentration) |

## 1. PUBMED

### *A.* Keywords

| Steps | Keywords | Results (# of studies) |
|---|---|---|
| **S1** | "pectus excavatum" OR "funnel chest" | 3,853 |
| **S2** | exercise OR treadmill OR bicycle OR cycle OR watt OR effort OR ergometer OR CPX OR CPET OR "cardio-respiratory" OR cardiorespiratory OR "cardio-pulmonary" OR cardiopulmonary | 2,158,965 |
| **S3** | O2 OR oxygen OR hemoglobin OR haemoglobin OR saturometer OR saturation OR SpO2 OR SaO2 OR arterial OR DAaO2 OR DA-aO "alveolo-arterial" OR "pulse oximetry" OR physiological | 1,221,414 |
| **S4** **(S1 AND S2 AND S3)** | ("pectus excavatum" OR "funnel chest") AND (exercise OR treadmill OR bicycle OR cycle OR watt OR effort OR ergometer OR CPX OR CPET OR "cardio-respiratory" OR cardiorespiratory OR "cardio-pulmonary" OR cardiopulmonary) AND (O2 OR oxygen OR hemoglobin OR haemoglobin OR saturometer OR saturation OR SpO2 OR SaO2 OR arterial OR DaaO2 OR DA-aO "alveolo-arterial" OR "pulse oximetry" OR physiological) | 48 |

### *B.* MeSH terms

| Steps | Keywords | Results (# of studies) |
|---|---|---|
| **S1** | Funnel chest | 3,107 |
| **S2** | exercise OR exercise test OR physical exertion | 650,614 |
| **S3** | oxygen OR hemoglobin* OR oxygen saturation | 1,170,052 |
| **S4** **(S1 AND S2 AND S3)** | ((Funnel chest) AND (exercise OR exercise test OR physical exertion)) AND (oxygen OR hemoglobin* OR oxygen saturation) | 39 |

Notes:

- Since inception
- In all fields
- All type of articles

## 2. SCIENCEDIRECT

### *A.* Keywords

| Steps | Keywords | Results (# of studies) |
|---|---|---|
| **S1** | "pectus excavatum" OR "funnel chest" | 3,530 |
| **S2** | exercise OR effort OR "cardio-respiratory" OR cardiorespiratory OR CPET | +1,000,000 |
| **S3** | hemoglobin OR oxygen | +1,000,000 |
| **S4**<br><br>**(S1 AND S2 AND S3)** | ("pectus excavatum" OR "funnel chest") AND (exercise OR effort OR "cardio-respiratory" OR cardiorespiratory OR CPET) AND (hemoglobin OR oxygen) | 508 |

### *B.* MeSH terms

No MeSH terms research available in this database.

N = 0

<u>Notes:</u>

- Since inception
- In all fields
- Only research articles and review articles

3. **CINAHL**

*A.* **Keywords**

| Steps | Keywords | Results (# of studies) |
|---|---|---|
| **S1** | "pectus excavatum" OR "funnel chest" | 576 |
| **S2** | exercise OR treadmill OR bicycle OR cycle OR watt OR effort OR ergometer OR CPX OR CPET OR "cardio-respiratory" OR cardiorespiratory OR "cardio-pulmonary" OR cardiopulmonary | 464,924 |
| **S3** | O2 OR oxygen OR hemoglobin OR haemoglobin OR saturometer OR saturation OR SpO2 OR SaO2 OR arterial OR DAaO2 OR DA-aO "alveolo-arterial" OR "pulse oximetry" OR physiological | 266,901 |
| **S4**<br><br>**(S1 AND S2 AND S3)** | ("pectus excavatum" OR "funnel chest") AND (exercise OR treadmill OR bicycle OR cycle OR watt OR effort OR ergometer OR CPX OR CPET OR "cardio-respiratory" OR cardiorespiratory OR "cardio-pulmonary" OR cardiopulmonary) AND (O2 OR oxygen OR hemoglobin OR haemoglobin OR saturometer OR saturation OR SpO2 OR SaO2 OR arterial OR DaaO2 OR DA-aO "alveolo-arterial" OR "pulse oximetry" OR physiological) | 10 |

*B.* **MeSH terms**

| Steps | Keywords | Results (# of studies) |
|---|---|---|
| **S1** | "Funnel chest" | 388 |
| **S2** | Exercise OR treadmills OR bicycle* OR ergomet* OR exertion OR "cardiorespiratory fitness" OR "exercise test" | 234,043 |
| **S3** | Oxygen OR hemoglobin* OR saturation OR "pulse oximet*" OR oximeter* OR physiological | 199,528 |
| **S4**<br>**(S1 AND S2 AND S3)** | ("Funnel chest") AND (Exercise OR treadmills OR bicycle* OR ergomet* OR exertion OR "cardiorespiratory fitness" OR "exercise test") AND (Oxygen OR hemoglobin* OR saturation OR "pulse oximet*" OR oximeter* OR physiological) | 5 |

Notes:

- Since inception
- In all fields
- All type of articles

## 4. SPORTDISCUSS

### *A.* Keywords

| Steps | Keywords | Results (# of studies) |
|---|---|---|
| **S1** | "pectus excavatum" OR "funnel chest" | 25 |
| **S2** | exercise OR treadmill OR bicycle OR cycle OR watt OR effort OR ergometer OR CPX OR CPET OR "cardio-respiratory" OR cardiorespiratory OR "cardio-pulmonary" OR cardiopulmonary | 398,599 |
| **S3** | O2 OR oxygen OR hemoglobin OR haemoglobin OR saturometer OR saturation OR SpO2 OR SaO2 OR arterial OR DAaO2 OR DA-aO "alveolo-arterial" OR "pulse oximetry" OR physiological | 96,064 |
| **S4**<br><br>**(S1 AND S2 AND S3)** | ("pectus excavatum" OR "funnel chest") AND (exercise OR treadmill OR bicycle OR cycle OR watt OR effort OR ergometer OR CPX OR CPET OR "cardio-respiratory" OR cardiorespiratory OR "cardio-pulmonary" OR cardiopulmonary) AND (O2 OR oxygen OR hemoglobin OR haemoglobin OR saturometer OR saturation OR SpO2 OR SaO2 OR arterial OR DaaO2 OR DA-aO "alveolo-arterial" OR "pulse oximetry" OR physiological) | 2 |

### *B.* MeSH terms

| Steps | Keywords | Results (# of studies) |
|---|---|---|
| **S1** | "Funnel chest" | 2 |
| **S2** | Exercise OR treadmills OR bicycle* OR ergomet* OR exertion OR "cardiorespiratory fitness" OR "exercise test" | 340,843 |
| **S3** | Oxygen OR hemoglobin* OR saturation OR "pulse oximet*" OR oximeter* OR physiological | 86,782 |
| **S4**<br>**(S1 AND S2 AND S3)** | ("Funnel chest") AND (Exercise OR treadmills OR bicycle* OR ergomet* OR exertion OR "cardiorespiratory fitness" OR "exercise test") AND (Oxygen OR hemoglobin* OR saturation OR "pulse oximet*" OR oximeter* OR physiological) | 0 |

Notes:

- Since inception
- In all fields
- All type of articles
