# Supplementary material for: Exercise impairment in patients with pectus excavatum? A scoping review of evidence and role of arterial content change during effort
Source: Physiol Rep. 2026 Jul 6;14(13):e71005. doi: 10.14814/phy2.71005 (PMC13338107; doi:10.14814/phy2.71005)
Supplement: Supplementary file 2 — Data S2. [file PHY2-14-e71005-s004.pdf]

General informations						Population			Measurement technique			Outcomes				Key results												Conclusion	
Authors	Year	Title	Country	Aims	Design	Adults (>18yo)	Both adults and youg	Young (<18yo)	Exercise capacity	DAV content	Spirometry/DLCO	Exercise capacity	Oxygen pulse	DAV content	Spirometry	Exercise capacity in %	Absolute exercise capacity	O2 pulse max %	O2 pulse max mL/beat	DAV content	FVC %	FVC L	FEV1 %	FEV1 L/s	TLC %	TLC L	Other results	Physiopathological hypothesis	Comments
Abu-Tair et al.	2018	Impact of Pectus Excavatum on Cardiopulmonary Function	Germany	The purpose of this prospective study was to evaluate the influence of the severity of dysmorphia on cardiopulmonary function by following a standardized protocol and to estimate which standardized magnetic resonance imaging (MRI)–derived respiratory status and index reflects the cardiopulmonary impairment most accurately.	Prospective cohort study	NA	Males : 18.8 yo (sd 7.7)/ Females : 16.1 yo (sd 4.7) | 99 participants (82 males and 17 females)	NA	CPET on treadmill	NA	NA	VO2max in % and mL/min/kg	O2 pulse in % and mL/beat	NA	NA	Males : 93.3% (sd 14.8), Females : 101.0% (sd 18.3)	Males : 43.3mL/kg/min (sd 6.6), Females : 36.6mL/kg/min (sd 6.0)	Males : 90.8% (sd 19.9), Females : 95.4% (sd 12.0)	Males : 13.5mL/beat (sd 3.4), Females : 8.8mL/beat (sd 1.5)	na	NA	NA	NA	NA	NA	NA	In 23 probands (23.2%), the VO2max% was less than 85% of the predicted value.	Reduced stroke volumes due to a decrease in retrosternal space, leading to impaired diastolic function of the right ventricle. In this case, the mechanism is presumably compensated by an increase in heart rate at the anaerobic threshold.	CPET protocol: According to this protocol, the test started with a speed of 2 km/h on a flat treadmill, increasing stepwise in increments of 0.5 km/h and 3% inclination every 90 seconds to amaximumincline of 21%. Beyond this inclination, only the speed continued to increase in increments of 0.5 km/h. The testing was stopped when the calculated target heart rate (ie, 220 – age in years) was reached or when complete exhaustion occurred, as indicated by the patient giving a hand sign. The end of the exercise test protocol consisted of a recovery phase on a flat treadmill at a speed of 2 km/h.
Al-Assiri	2009	Operative innovation to the "Nuss" procedure for pectus excavatum: operative and functional effects	Canada	Our objective is to describe the perioperative and long-term physiologic consequences of this technical modification.	Comparative study	NA	SN: 13.5 yo (sd 1.9)/ MN group : 15.0 yo (sd 4.1) | Nb of participants: 30 (15 males and 15 females)	NA	CPET on treadmill and cycloergometer	NA	Spirometry	VO2max in % and VO2/kg in L/kg·min	O2 pulse in %	NA	FVC in % and L, FEV1 in % and L, TLC in % and L	SN: 68.40% (sd 9.99)/ MN: 72.00% (sd 19.75)	SN: 32.81 L/kg·min (sd 6.65), MN: 34.51 L/kg·min (sd 6.97)	SN: 73.47% (sd 10.27), MN: 76.00% (sd 19.38)	NA	NA	SN: 87.73% (sd 14.37), MN: 89.53% (sd 0.94)	SN: 3.27L (sd 0.94), MN: 3.35L (sd 1.15)	SN: 78.13% (sd 10.41), MN: 79.27% (sd 18.91)	SN : 2.72L/s (sd 0.78), MN : 2.75L/s (sd 0.94)	SN: 95.40% (sd 15.78), MN: 94.33% (sd 18.29)	SN: 4.55L (sd 1.28), MN: 4.60L (sd 1.35)	NA	NA	CPET protocol: A 10 or 25-W protocol was used depending on the patient's age (Sensormedics Corp, Yorba Linda, Calif) [15]. Subjects exercised to the point of fatigue or when they demonstrated an increase in carbon dioxide output without further increase in oxygen uptake (anaerobic threshold). Subjects were encouraged to continue exercise until this threshold was reached.
Borowitz et al.	2003	Pulmonary function and exercise response in patients with pectus excavatum after nuss repair	USA	The purpose of the present investigation has been to find the hernodynamic explanation for the influence of body position on the physical working capacity in cases with funnel chest.	Prospective cohort study	NA	NA	Mean age: 13.4 yo (sd 3) | 10 participants, all boys	CPET on cycloergometer	NA	Spirometry	VO2max in % and mL/min	NA	NA	FVC in % and L, FEV1 in % and L, TLC in % and L	85% (range= 52 - 106)	1882 mL/min (range = 1011 - 2621)	NA	NA	NA	91% (range= 61 - 108)	3.06L (range= 1.25 - 4.35)	91% (range= 65 - 112)	2.65L/s (range= 1.12 - 3.88)	91%	3.70L	NA	NA	CPET protocol: exercise measurements were made for 30 seconds at the end of each 2-minute work level during exercise. A ramping workload (weight in kilograms 0.3 watts) was imposed over each minute during exercise with a minimum of 10 watts/min and maximum of 20 watts/min ramp. The test was discontinued if the patient's systolic blood pressure exceeded 200 mm Hg; diastolic blood pressure exceeded 100 mm Hg; heart rate was consistently over 200 beats per minute; a cardiac dysrythmia was identified; the patient had severe dyspnea, chest pain, or wheezing; or until the point that the patient could no longer continue.
Cahill et al.	1984	A summary of preoperative and postoperative cardiorespiratory performance in patients undergoing pectus excavatum and carinatum repair	USA	Hypothesis: We hypothesized that placement of a substernal bar in the first stage of the Nuss repair will not adversely affect pulmonary and exercise function.	Prospective cohort study	NA	NA	Mean age: 10.9 yo (range= 6-17) | 14 PEx participants (and 5 pectus carinatum)	CPET on cycloergometer	NA	Spirometry	VO2max in mL/kg/min	NA	NA	FVC in L and TLC in L	NA	1.26 mL/kg/min (sd 0.44)	NA	NA	NA	NA	2.48L (sd 0.96)	NA	NA	NA	3.21L (sd 1.12)	NA	Patients with restrictive pulmonary disorders, such as severe pectus excavatum deformity, may not be able to increase ventilation because of restriction in chest wall mechanics.	CPET protocol: Progressive work exercise protocol with a cycle ergometer was used to evaluate maximal work capacity. The subject was seated on the cycle ergometer for a five minute rest period, then instructed to pedal at a constant rate (Fig 1). The work load was increased in increments of 100 kilpounds/meter/minute (KPM) each minute until the subject could no longer maintain the same speed despite active encouragement.
Cavestri et al.	2010	Exploration fonctionnelle à l'exercice des patients présentant un pectus excavatum	France	Le but de cette étude était d'analyser le comportement à l'exercice d'une série de patients présentant un PE, et de déterminer l'éventuel impact de l'épreuve fonctionnelle d'exercice (EFX) sur la décision médicale.	Cross-sectional study	NA	Mean age: 25.9 yo (sd 11.8) | 32 participants (25 males and 7 females)	NA	CPET on cycloergometer	Saturation and blood gas analysis	Spirometry and DLCO	VO2max in % and mL/kg/min	O2 pulse in %	Saturation + blood gas analysis	FVC in %, FEV1 in %, DLCO in % and mL/min/mmHg	78.6% (sd 22.1)	33.8 mL/kg/min (sd 10.4)	85.2% (sd 22.7)	NA	No objective results for SpO2. DaAO2max : 25.0mmHg (sd 14.7)	99% (sd 17)	NA	96.6% (sd 17.1)	NA	NA	NA	Un FOP était mis en évidence chez 28% des patients. Vingt patients présentaient une aptitude aérobie basse inférieure à 84%. Le gradient alvéolo-artériel en O2 était normal au pic de l'exercice. Toutefois des valeurs élevées du gradient alvéolo-artériel en O2 (> 35 mmHg au pic) étaient observés chez cinq des 32 patients. DLCO % : 85 (sd 22.4) | DLCO/VA : 95.6 (sd 13.7)	Le déconditionnement pourrait expliquer en partie la limitation à l'exercice. Cependant, Malek et al. Ont montré qu'il existait une diminution de l'aptitude aérobie chez des patients atteints de PE pratiquant une activité sportive régulière (trois heures par semaine en moyenne), ce qui laisse supposer que la déformation en elle-même est susceptible d'induire une diminution de l'aptitude aérobie. Nos données viennent confirmer cette hypothèse.	CPET protocol: L'EFX était triangulaire avec une période d'échauffement à 20 watts de trois minutes suivie d'un exercice incrémental réalisé selon un protocole en rampe de 10, 20 ou 30 watts par minute, selon la puissance attendue, poursuivi jusqu'à épuisement .
Das et al.	2019	Improvement of cardiopulmonary function after minimally invasive surgical repair of pectus excavatum (Nuss procedure) in children	USA	We studied the effect of pectus excavatum on cardiopulmonary response to exercise before and after repair, taking into consideration the severity of pectus excavatum (Haller index by computed tomography [CT] scan) to determine if there was any improvement in cardiopulmonary function.	Prospective cohort study	NA	NA	Mean age: 12.9 yo (sd 3.6) | 24 participants	CPET on treadmill	Saturation and blood gas analysis	Spirometry	VO2max in % and mL/kg/min	O2 pulse in % and mL/beat	Saturation at rest and peak exercise	FVC in % and L, FEV1 in % and L	62% (sd 25)	32 mL/kg/min (sd 13)	76% (sd 24)	9 mL/beat (sd 4)	No objective data were reported	85% (sd 13)	3.2L (sd 1.1)	85% (sd 11)	2.65L/s (sd 0.9)	NA	NA	NA	The improvement of maximum oxygen consumption in our study after surgical repair appears to be due to increase in stroke volume during exercise, and our results are similar to other published studies	CPET protocol: Cardiopulmonary exercise tests utilizing treadmill per Bruce protocol
Dupuis et al.	2024	Impact of pectus excavatum on pulmonary function and exercise capacity in patients treated with 3D custom-made silicone implants	France	The primary objective of this study was to investigate the impact of PE on pulmonary function test (PFT) and exercise capacity.	Prospective study	NA	Mean age: 28 yo (sd 11.3) | 60 participants (40 males et 20 females)	NA	CPET on cycloergometer	NA	Spirometry	VO2max in % and mL/min/kg	O2 pulse in % and mL/beat	NA	FVC in %, FEV1 in %, TLC in %	87% (sd 13)	33.4 mL/min/kg (sd 6.1)	92% (sd 15)	12.2 mL/beat (sd 3)	NA	96.4% (sd 16)	NA	99.7% (sd 16)	NA	98.5% (sd 12.6)	NA	patients with ventilatory limitations (n = 3, ventilatory reserve < 15%): 1 patient had a respiratory muscle dysfunction; one had an obstructive lung disease at rest, and one had no ventilatory trouble at rest nor respiratory muscle dysfunction. No cardiac limitation was reported.	Other studies suggest PE may result in a decreased capacity to increase systolic ejection volume during exercise, due to heart compression [24]. Compression of the right heart cavity could contribute to mitral valve prolapse according to some studies, an hypothesis supported by a higher prevalence of valvular disease in patients with PE.	CPET protocol: The protocol included 3 minutes of warm-up and then an increase in rate until maximal tolerance according to the theoretical maximal power.
Eldredge et al.	2025	Cardiopulmonary Impact of the Minimally Invasive Repair of Pectus Excavatum in Pediatric patients: A Prospective Pilot Study	USA	The primary objective of this project was to determine the impact of the minimally invasive repair of PE (MIRPE) on cardiopulmonary function during exercise in pediatric patients and to determine patient factors that impact changes in cardiopulmonary performance following MIRPE.	Prospective cohort observational study	NA	NA	Mean age: 15.28 yo (sd 1.5) | 81 participants (72% males and 28% females)	CPET on treadmill	NA	Spirometry	VO2max in % and VO2/kg in mL/kg/min	O2 pulse in % and mL/beat	NA	FVC in %, FEV1 in %	75.9 % (sd 16.9)	35.0 mL/kg/min (sd 7.4)	79.1% (sd 18.4)	10.3 mL/beat (sd 3.2)	NA	80.9% (sd 17.4)	NA	85.0% (sd 10.5)	NA	NA	NA	NA	The improvement in the stroke volume response may be secondary to the anatomic relief of cardiac compression due to the PE deformity following MIRPE. Jaroszewski et al. conducted a study measuring left ventricular stroke volume in adult patients at the time of MIRPE prior to sternal elevation and at the time of pectus bar removal using intraoperative transesophageal echocardiograms (TEE).	CPET protocol: CPET analyses were conducted using an MGC Ultima CardioO2 metabolic cart (Medical Graphics, St Paul,MN) with the same treadmill protocol as their pre-MIRPE study. During exercise, patients were encouraged to reach a point of exhaustion by running.
Ghory et al.	1989	Cardiac performance in children with Pectus excavatum	USA	To do this we studied the cardiovascular response during maximum upright graded exercise testing in preoperative patients with pectus excavatum aged 6 to 16 years. This response was compared with that of normal control patients.	Cross-sectional study	NA	NA	Mean age for PEx group : 10.2 yo (sd 4.2) | 28 participants (14 PEx and 14 controls)	CPET on cycloergometer	NA	NA	VO2max in % and mm/min	NA	NA	NA	91% (sd 12)	1.749mm/min (sd 854)	NA	NA	NA	NA	NA	NA	NA	NA	NA	Arterio-veinous différence: 12 (sd 1)	NA	CPET protocol: cycle ergometer and the James protocol) The children's ages ranged from 5 to 16 years (Table 1). They exercised to exhaustion and a maximal level of intensity as reflected by their percent of predicted maximal oxygen uptake (>75%).
Haller and Loughlin	2000	cardiorespiratory function is significantly improved following corrective surgery for severe pectus excavatum	USA	To updates previous work from our group which described the approach employed at The Hop-kins Children's Center for diagnosis and treatment of adolescents with severe pectus excavatum. We are summarizing Our recent experience with measurements of exercise pulmonary and cardiac functions.	Comparative study	NA	NA	Mean age : 16 yo (sd 3) | 36 participants	CPET on treadmill	Saturation	Spirometry	VO2max in mL/kg/min	O2 pulse in mL/beat	Continue monitoring of sat	FVC in %, FEV1 in %, TLC in %	NA	40 ml/kg/min (sd 7)	NA	11.5mL/beat (sd 3.7)	No objective data were reported	81% (sd 14)	NA	79% (sd 14)	NA	89% (sd 17)	NA	NA	The most likely reason for the increased oxygen pulse is an increased stroke volume due to reliefo f cardiac compression by the sternal deformity.	CPET protocole : In all exercise studies, data were initially measured for three minutes at rest. Treadmill speed was then increased each minute until a speed of 7 kilometers per hour was reached, following which the grade was increased by 2.5% each minute, until a grade of 20% was reached. If the subject continued to exercise, the treadmill speed was then increased to 8 kilometers per hour. The study continued until the subject could no longer exercise. Recovery data were collected for three minutes.
Jaroszewski et al.	2022	cardiopulmonary outcomes after the nuss procedure in pectus excavatum	USA	This study evaluates the impact of pectus excavatum on the cardiopulmonary function of adult patients before and after a modified minimally invasive repair.	Retrospective cohort study	Mean age : 31.0 yo (sd 9.8) | 392 patients (267 males and 125 females)	NA	NA	CPET on cycloergometer	Saturation	NA	VO2max in % and mL/kg/min	O2 pulse in % and mL/beat	Continue monitoring of sat	NA	73.6% (sd 15.8)	26.5mL/kg/min (sd 6.2)	86.0% (sd 17.3)	11.7 mL/beat (sd 3.5)	No objective data were reported	NA	NA	NA	NA	NA	NA	67.9% had an abnormal VO2max score (<80%). 34.4% had an O2 pulse <80%.	NA	CPET protocol: Incremental exercise tests were performed using a calibrated electromagnetically upright cycle (Corival, Lode, Groningen, The Netherlands) with a non-invasive, photo acoustic gas-rebreathing analyzer (Ultima Series, MGC Diagnostics Corporation, Saint Paul, MN). A standardized 1-minute step protocol at 25 W/min was used. The participants were asked to pedal at a steady pace of 60 rpm. Maximal incremental exercise was performed until exhaustion.
Kelly et al.	2013	multicenter study of pectus excavatum, final report: complications, static/exercise pulmorary function, and anatomic outcomes	USA	To examine the safety, efficacy, and complications of surgical repair of pectus excavatum.	Multicenter prospective cohort study	NA	NA	Mean age : patients who completed the whole study 13.60 yo (sd 3.26), patients lost 14.37 yo (sd 3.50) | 327	CPET on both cycloergometer or treadmill depending on the center	NA	Spirometry	VO2max in L/min	O2 pulse in mL/beat	NA	FVC in %, FEV1 in %, TLC in %	NA	3.18L/min (sd 3.33)	NA	13.58 mL/beat (sd 3.53)	NA	87.9% (sd 13.0)	NA	86.6% (sd 13.0)	NA	94.3% (sd 12.8)	NA	NA	We measured the oxygen pulse during peak or maximum exercise and have interpreted this increase in O2 pulse to reflect an increase in stroke volume.	CPET protocol: difference between centers
Maagaard et al.	2013	Normalized cardiopulmonary exercise function in patients with pectus excavatum three years after operation	Danmark	To further clarify the impact of surgical correction of the thoracic wall on cardiopulmonary function at rest and during exercise.	Single center prospective controlled study	NA	NA	Mean age : 15.5 yo (sd 1.7) for PEx group, and 15.0 yo (sd 1.9) for control group | 49 patients (10 females and 39 males) and 26 control subjects (10 females and 16 males)	CPET on cycloergometer	Saturation	Spirometry	VO2/kg in mL/min/kg	NA	Continue monitoring of sat	FVC in %, FEV1 in %	NA	26 ml/min/kg (sd 7,1)	NA	NA	No objective data were reported	92% (sd 14)	NA	86% (sd 13)	NA	NA	NA	NA	further accentuates the association between postoperative improved function and an increase in cardiac (stroke) volume during exercise	CPET protocol: The participants pedaled at a steady pace of 60 rpm with incremental workload (steps of 30W) until exhausted and no longer in position to continue despite encouragement from the research team. A sub-maximal exercise effort was achieved with a heart rate of approximately 80% of expected maximum value as well as subjective evidence of fatigue

Malek et al.	2003	Ventilatory and cardiovascular responses to exercise in patients with pectus excavatum	USA	To assess whether parameters of aerobic fitness such as Volinas would be lower than the reference values specific for each patient, despite regular participation in exercise.	Cross-sectional study	NA	Mean age : 23.6 yo (sd 8.9) | 21 participants (15 males and 6 females)	NA	CPET on cycloergometer	Saturation	Spirometry and DLCO	VO2max in % and L/min	O2 pulse in % and mL/beat	Continue monitoring of sat	FVC in % and L, FEV1 in % and L, TLC in % and L, DLCO in mL/min/mmHg	75% (sd 19)	2.14L/min (sd 0.75)	86%	12.4 mL/beat (sd 3.9)	95.5% (sd 2.1)	85%	4.26L	87%	3.53L/s	92%	5.83L	DLCO : 2>8.6mL/min/mmHg (sd 8.15)	NA	CPET protocol: The work rate increment was judged for each individual subject by considering age, gender, heigth, weight and patient-reported degree of functional impairment with the intention of obtaining an exercise phase of 8 to 12 min before exhaustion.
Morshuis et al.	1994	Exercise cardiorespiratory function before and one year after operation for pectus excavatum	Netherlands	To assess the degree of eventual cardiorespiratory impairment by pectus excavatum and to investigate the value of surgical correction.	Prospective cohort study	NA	Mean age : 17.9 yo (sd 5.6) | 35 participants (28 males and 7 females)	NA	CPET on cycloergometer	Blood sample from brachial arteris and saturation	Spirometry and DLCO	VO2/kg in mL/min/kg	O2 pulse in mL/beat	Continue monitoring of sat + arterial PaO2 measure every 3min	FVC in % and L, FEV1 in % and L, TLC in % and L, DLCO unit not reported	NA	26.8 mL/min/kg (sd 6.1)	NA	8.2 mL/beat (sd 2.6)	PaO2 (between rest and exercise) : -1.0 (sd 1.6) | SpO2 : no objective data were reported	79.7% (sd 16.2)	3.877 L (sd 1.475)	83.8% (sd 17.8)	3.214 L/s (sd 1.177)	86.0% (sd 14.3)	5.081 L (sd 1.683)	DaAO2 : 3.13 (sd 1.15) | Unlike normal subjects, PaO2 do not increase during effort. No change in blood oxygen before and after operation. DLCO : no objective data were reported	Castile, Staats, and Westbrook'? found that V02 during exercise exceeded predicted values at greater work loads in fivepatients with symptomatic pectus excavatum, whereas two patients without symptoms did not show the same process. These investigators suggested that these findings in patients with symptomatic deformities were caused by decreased chest wall compliance and an increased work of breathing. Because the oxygen uptake at rest was unchanged, the significant increase in Vo2 after operation together with increased acidification at exercise may suggest that more muscle activity is needed at maximal exercise that does not contribute to external work load, which may indicate an increased activity of the ventilatory musculature	CPET protocol: The patient pedaled at 60 to 70 rpm with I-minute incremental workload intervals. The increment of the workloads was based on 10% of the patient's expected performance. All patients were encouraged to exercise to exhaustion.
Neviere et al.	2011	Cardiopulmonary response following surgical repair of pectus excavatum in adult patients	France	The present study aims at assessing whether surgical repair would improve exercise tolerance in a large population of adult patients with severe PE.	Prospective cohort study	Mean age of operated patients : 27.11 yo (range= 18 - 62) | 70 participants (9 females and 61 males)	NA	NA	CPET on cycloergometer	NA	Spirometry and DLCO	VO2max in % and mL/kg/min	O2 pulse in % and mL/beat	NA	FVC in % and L, FEV1 in % and L, TLC in % and L, DLCO in mL/min/mmHg	77% (sd 2)	34.9 mL/kg/min (sd 7.5)	91% (sd 12)	13.2 mL/beat (sd 3.1)	NA	92%	4.64 L (sd 0.89)	92%	3.91 L/s (sd 0.81)	95%	6.75L (sd 1.20)	DaAO2 : 2.53mmHg (sd 0.93) | DLCO : 29.0mL/min/mmHg (sd 4.6).	This is in line with previous studies showing significant decreases in O2 uptake and O2 pulse in PE patients, which was not explained by physical deconditioning. These findings suggest that, in PE patients, cardiac filling and cardiac output might be limited by the result of compression by the displaced sternum on the right heart chambers, thereby limiting increase of stroke volume. At exercise, these changes might impede further the heart's ability to increase the stroke volume, to meet increased metabolic demands. These findings suggest that PE repair could improve cardiac filling and cardiac output at maximal workload as a result of the chest's anteroposterior diameter increase.	CPET protocol: Incremental exercise tests were performed on a calibrated electromagnetically braked cycle ergometer (ER-900; Jaeger, Hochberg, Germany), using a 1-min step protocol at 10 W min−1 until exhaustion [10]. Maximal incremental exercise was performed with the subjects maintaining a pedaling frequency of 60 5 revolutions of the crank per min. Peak exercise was defined as the highest work level reached during the incremental exercise test.
O'keef et al.	2013	Longer term effects of closed repair of pectus excavatum on cardiopulmonary status	Canada	The objective of this study was to compare cardiopulmonary function and subjective evaluation of appearance and exercise tolerance pre-bar insertion with post-bar removal.	Prospective cohort study	NA	NA	Mean age of lost participants : 13 yo (sd 3.4), mean age of followed patients : 13.9 yo (sd 2.3) | 130 participants (67 who only completed the whole follow up, and 63 lost)	CPET on cycloergometer or treadmill	Saturation	Spirometry and DLCO	VO2max in % only	O2 pulse in %	Continue monitoring of sat	FVC in %, FEV1 in %, TLC in %	Lost patients : 75.8% (sd 18.2) | Followed patients : 70.1% (sd 15.0)	NA	Lost patients : 83.7% (sd 18.7) | Followed patients : 75.8% (sd 14.4)	NA	No objective data were reported	Lost patients : 99.6 (sd 18.5) | Followed patients : 91.2 (sd 18.6)	NA	Lost patients : 90.8% (sd 15.8) | Followed patients : 81.1% (sd 17.0)	NA	Lost patients : 102.6% (sd 17.8) | Followed patients : 99.2% (sd 16.5)	NA	NA	We believe in part these results highlight the limitations of extrapolating cardiopulmonary assessment done at rest to the physiologic effects with exercise. The subjective improvement after repair is classically described by the patients as an improvement in maximum exercise capacityPectus excavatum creates a fixed restriction in the chest volume which affects the heart, decreasing stroke volume and hence cardiac output, especially at high load.	CPET protocol: A 10 or 25 W protocol was used depending on the patient's age
Oleksak et al.	2022	Correlation of anthropometric index and cardiopulmonary exercise testing in children with pectus excavatum	Slovakia	To use cardiopulmonary exercise testing (CPET) to evaluate the usability of the anthropometric index (AI) in patients with pectus excavatum as a marker of functional impairment caused by chest deformity.	Cross-sectional study	NA	Mean age: 15.3 yo (sd 2.9) | 32 patients (28 males and 4 females)	NA	CPET on treadmill	NA	NA	VO2peak in z-score	O2 pulse in z-score	NA	NA	NA	z-score = −1.51 (sd 1.11)	NA	z-score = −1.21 (sd 1.10)	NA	NA	NA	NA	NA	NA	NA	NA	According to Malek et al. (2006) sternal compression reduces the sternum volume, leading to a reduction in maximal oxygen consumption during exercise, exercise tolerance, tidal volume and vital capacity, all of which reduce body endurance and cause dyspnoea and compensatory tachypnoea during exercise. Reduced oxygen supply to the working muscle as a consequence of reduced venous return to the right atrium also contributes to the reduced physical fitness of patients with PE.	CPET protocol: An individualised load protocol was used to achieve the maximum tolerated load between 8 and 12 min. Prior to the exercise, a manoeuvre was performed to determine the inspiratory capacity during calm breathing, and subsequently the inspiratory capacity was paired with the tidal volume. | Data are presented as Z scores
Quigley et al.	1996	Cardiorespiratory function before and after corrective surgery in pectus excavatum	USA	To determine whether pectus excavatum (PE) results in cardiopulmonary abnormalities, and whether surgical repair results in improvement.	Comparative study	NA	Mean age : 16 yo (sd 3) | 36 patients (32 males and 4 females)	NA	CPET on treadmill	Saturation	Spirometry	VO2max in mL/kg/min	O2 pulse in mL/beat	Continue monitoring of sat	FVC in %, FEV1 in %, TLC in %	NA	40 mL/kg/min (sd 7)	NA	11.8 mL/beat (sd 3.3)	Minimal SaO2 : 96% (sd 2) | Resting SpO2 : 97% (sd 1)	81% (sd 14)	NA	79% (sd 14)	NA	89% (sd 17)	NA	NA	The most likely reason for the increased 02 pulse postoperatively is an increased stroke volume because of relief of cardiac compression by the sternal deformity.	CPET protocol: All exercise studies were performed with the same equipment, conducted by the same supervisors (P. M. Q. and K. L. J.) under the same conditions. Subjects were encouraged to exercise for as long as possible. Data were initially measured for 3 minutes with the subjects at rest. Treadmill speed was then increased each minute until a speed of 7 km/hr was reached, after which the grade was increased by 2.5% each minute until a grade of 20% was reached. If the subject continued to exercise, the treadmill speed was then increased further to 8 kin/hr. The study continued until the subject could exercise no longer. Recovery data were collected for 3 minutes. The same protocol for increase in workload was used for all subjects and was designed in an attempt to have subjects reach near-maximal oxygen consumption in approximately 8 to 12 minutes Data provided are those for both PEx, pectus carinatums and arcuatums.
Ravanbakhsh et al.	2022	Sex Differences in Objective Measures of Adult Patients Presenting for Pectus Excavatum Repair	USA	The purpose of this study was to report our experience with differences between adult male and female patients who underwent surgical repair for pectus excavatum.	Retrospective study	Females mean age : 35.0 yo (sd 11.8), males mean age : 32.4 yo	NA	NA	CPET	NA	Spirometry	VO2max in % (predicted)	O2 pulse in %	NA	FEV1 in %, TLC in %	Females: 77 % (sd 16.9) | Males: 72.2% (sd 15.7)	NA	Females: 91% (sd 18.6) | Males: 84.4% (sd 17.0)	NA	NA	NA	NA	Females: 81.6% (sd 14.3) | Males: 83.2% (sd 18.1)	NA	Females: 89.5% (sd 13.7) | Males: 93.9% (sd 16.8)	NA	NA	NA	No detail provided
Sanjurjo et al.	2024	Unsophisticated assessment of the cardiopulmonary function in patients with pectus excavatum using the six minute walk test	Argentina	We therefore sought to explore whether the maximum predicted walking distance is decreased among patients with PEX.	Retrospective study	NA	Mean age : 17.8 yo (sd 6.7), range= 11 - 36 yo | 43 participants (38 males and 5	NA	6MWT	transcutaneous oximetry	NA	Distance to 6MWT	NA	transcutaneous oximetry before and after test	NA	NA	NA	NA	NA	Baseline oxygen: between 97.4% (sd 1.2) | Final oxygen: 96.4% (sd 2.1)	NA	NA	NA	NA	NA	NA	The mean maximum distance walked was significantly lower than the predicted distance (600.8 ± 67.6 metres vs. 729.8 ± 67.5 metres, p < 0.0001), with a mean negative	NA	Surprisingly, authors mention using TCpO2 for oxymetery, but data are provided as saturation in percentage.
Satur et al.	2021	Can categorised values of maximal oxygen uptake discriminate patterns of exercise dysfunction in pectus excavatum: a prospective cohort	UK	We undertook a retrospective study to examine whether subgroup analysis of CPET data allowed delineation of differing patterns of exercise function among patients with PE.	Retrospective study	NA	Mean age : 28 yo (sd 6.6) | 70 participants (60 males and 10 females)	NA	CPET on cycloergometer	Saturation	Spirometry	VO2max in %	O2 pulse in %	Continue monitoring of sat	FVC in %, FEV1 in %	78.0% (sd 13.7)	NA	88.9% (sd 16.3)	NA	No objective data were reported	91.8% (sd 11.9)	NA	92.0% (sd 12.8)	NA	NA	NA	NA	The results contradict the view that patients with PE are 'deconditioned', more bluntly described, 'lazy' with a lack of physical motivation. The mechanistic cause of exercise dysfunction and subsequent improvement following surgical treatment however, remain unproven.	CPET protocol: CPET used a pretest 3 min rest period followed by a 3 min warm-up undertaking unloaded pedalling at 60–70 rotations per minute. Subsequent exercise protocol used a continuous uniform increase in work rate and patients were encouraged to exercise to exhaustion. Individual test protocols were selected to produce 8–12 min of exercise during the ramp phase.
Sigalet et al.	2007	Long term cardiopulmonary effects of closed repair of pectus excavatum	Canada	This study examined the effects at 3 months post-bar removal of closed repair of pectus on pulmonary function, exercise tolerance and cardiac function.	Prospective cohort study	NA	NA	Mean age : 13.2 yo (sd 2.1) | 26 participants	CPET on cycloergometer or treadmill	Saturation	Spirometry and DLCO	VO2max in % and VO2/kg in mL/kg/min	O2 pulse in %	Continue monitoring of sat	FVC in % and L, FEV1 in % and L, TLC in % and L	70.8%(sd 11)	34.1 mL/kg/min (sd 6.1)	77.1% (sd 9.5)	NA	No objective data reported	89.5% (sd 18.6)	3.28L (sd 1.04)	78.4% (sd 16.0)	2.67L/s (sd 0.81)	95.3% (sd 16.0)	4.47L (sd 1.22)	NA	It is due to a combination of improved efficiency of ventilation, and increased stroke volume (and secondarily cardiac output) at exercise. The perceived shortness of breath with intense exercise, which patients describe preoperatively is	CPET protocol: A 10 or 25 W protocol was used depending on the patients age
Swanson et al.	2012	Correlating Haller Index and cardiopulmonary disease in pectus excavatum	USA	We compared patterns of cardiopulmonary function, and their relationship with HI, in patients with pectus excavatum.	Retrospective study	NA	NA	Mean age : 15.2 yo (sd 0.3) | 87 participants (80% males and 20% females)	CPET on cycloergometer	Saturation	Spirometry	VO2max in %	O2 pulse in %	Continue monitoring of sat	FVC in %	86.3% (se 1.7)	NA	98.4% (se 1.7)	NA	No objective data were reported	89.8% (se 1.4)	NA	NA	NA	NA	NA	NA	One hypothesis is that the right ventricle may experience compression earlier and with greater magnitude compared with the lungs, with resultant decreased right ventricular filling, because of its immediately substernal location and smaller relative volume.	CPET protocol: Exercise was performed on a cycle ergometer using a mouthpiece while metabolic data were collected. Three minutes of initial rest were followed by 3 minutes of unloaded pedaling at 60 rpm, then symptom-limited exercise, and 3 minutes of recovery at minimal workload.
Tang et al.	2012	Improved cardiopulmonary exercise function after modified Nuss operation for pectus excavatum	Danmark	This study questions whether minimally invasive operation for pectus excavatum in a teenager influences the cardiopulmonary function at rest and/or during exercise.	Comparative study	NA	NA	Mean age : 15.5 yo (sd 1.7), participants = 49 | mean age controls : 15.0 yo (sd 1.9), Controls = 26	CPET on cycloergometer	Saturation	Spirometry	VO2/kg in mL/kg/min	NA	Continue monitoring of sat	FVC in %, FEV1 in %	NA	26 mL/kg/min (sd 6.0)	NA	NA	No objective data were reported	96% (sd 14)	NA	87% (sd 13)	NA	NA	NA	NA	NA	CPET protocol: Participants cycled in the upright position to exhaustion, with a progressive protocol of 3 min work stages and 30 W increments. Prior to increasing work load a new breath test was made. Breath test could not be made at shorter intervals than 3 min due to the recommended time needed for clearance of gas particles. Pedalling cadence was 60 rpm. After instruction and 2–3 rebreathing procedures for practicing, the patient was placed on the bicycle and equipped with a pulse oxymeter.
Udholm et al.	2016	Cardiac function in adults following minimally invasive repair of pectus excavatum	Danmark	The aim of this study was to assess cardiac and pulmonary function at rest and during exercise and compare preoperative results with results 1 year after undergoing the Nuss procedure.	Single center prospective controlled study	Mean age : 32 yo | 19 participants (6 females and 13 males)	NA	NA	CPET on cycloergometer	NA	Spirometry	VO2/kg in mL/kg/min	NA	NA	FVC in %, FEV1 in %	NA	30.4 mL/kg/min (sd 6)	NA	NA	NA	90% (sd 9)	NA	89% (sd 10)	NA	NA	NA	NA	NA	CPET protocol: The participants pedalled at a steady pace of 60 rpm with incremental workload (steps of 30 W)until exhausted and no longer capable of continuing despite encouragement from the research team. A submaximal exercise effort was achieved with a heart rate of 80% of expected maximum value as well as subjective evidence of fatigue. Spirometry data is provided for n=13.

Wynn et al.	1990	Exercise cardiorespiratory function in adolescents with pectus excavatum : observations before and after operation	USA	Therefore, we prospectivelyevaluated two groups of adolescent patients with pectus excavatum: one group elected to have surgical correction and the other did not.	Prospective controlled study	NA	NA	Mean age of operated participants: 13.8 yo | Mean age of non-operated participants: 14 yo (n=4) | 13 participants (11 males and 2 females) (age range= 10 - 16)	CPET on cycloergometer	Saturation	Spirometry and DLCO	Vo2max in mL/kg/min	NA	Continue monitoring of sat	FVC in % and L, FEV1 in % and L, TLC in % and L, DLCO unit was not reported	NA	Operated group: 36.1 mL/kg/min (sd 4.4) | Non operated group: 41.2 mL/kg/min (sd 7.3)	NA	NA	No saturation drop during exercise (no objective data were reported)	Operated group: 99.0% (sd 18.0)(n=7) | Non operated group: 117.0% (sd 16.5)(n=4)	Operated group: 3.2L (sd 1.1)(n=7) | Non operated group: 4.2L (sd 1.3)(n=4)	Operated group: 99.0% (sd 25.0)(n=7) | Non operated group: 104.6% (sd 9.0)(n=3)	Operated group: 2.8 L/s (sd 1.0)(n=7) | Non operated group: 3.0L/s (sd 0.5)(n=3)	Operated group: 80.3% (sd 6.6)(n=7) | Operated group: 89.4% (sd 8.7)(n=3)	Operated group: 4.53L (sd 1.2)(n=7) | Non operated group: 5.5L (sd 1.5)(n=3)	Objective data regarding DLCO were not reported	We found no apparent abnormalities of cardiac output and stroke volume responses during exercise. Also, there was no difference in stroke volume either at rest or during exercise postoperatively compared with preoperatively.	CPET protocol: Exercise testing was performed on a previously calibrated, electronically braked cycle ergometer (Siemens-Elema 380B, Siemens Medical Systems Inc., Iselin, N.J.); the patient pedaled at 60 to 70 rpm with 3-minute incremental work-load intervals. One of three combinations of work loads was used based on the patient's body surface area.l" All individuals were encouraged to exercise to exhaustion. The degree of effort was assessed subjectively by one of the investigators during the test and objectively by comparing the patient's maximal heart rate, oxygen uptake, and work performance with established normal values.
Zens et al.	2022	The Severity of Pectus Excavatum Defect Is Associated With Impaired Cardiopulmonary Function	USA	The primary goals of this research were to evaluate whether pectus excavatum is associated with cardiopulmonary dysfunction and establish whether a correlation exists between the severity of the deformity and the degree of cardiopulmonary dysfunction.	Cross-sectional study	NA	Mean age: 15.2 yo (sd 3.95) | 345 participants (81% males and 19% females)	NA	CPET on cycloergometer	NA	Spirometry	VO2max in %	O2 pulse in %	NA	FVC in %, FEV1 in %, TLC in %	89.1% (sd 16.5)	NA	91.1% (sd 17.7)	NA	NA	96.35% (sd 13.1)	NA	92.34% (sd 12.3)	NA	99.13% (sd 11.55)	NA	78% of patients presented a right ventricle compression. 30% had a reduced VO2max (<80%) and 27% had a reduced O2 pulse (<80%).	Despite compression of the right side of the heart, LV function was also affected, with LVEF <0.55 in 22% of patients and an LVEF Z-score of < [illegible]2 in 18% of patients, highlighting the potential for pectus excavatum to have a detrimental effect on biventricular cardiac function.	CPET protocol: CPET was performed on a cycle ergometer (ViaSprint 150p, Ergoline) using a ramping protocol.
Zhao et ak.	2000	Why is exercise capacity reduced in subjects with pectus excavatum?	Israel	The purpose of this study was to test this hypothesis by comparing SV and oxygen uptake during supine and sitting exercise in patients with PE and in healthy control subjects.	Comparative study	NA	Mean age of experimental group: 19 yo(sd 6), 13 participants (2 females and 11 [illegible]	NA	CPET on cycloergometer	NA	Spirometry and DLCO	VO2max in mL/min	O2 pulse in mL/beat	NA	FVC in %, FEV1 in %, TLC in %	NA	In sitting position : 1480mL/min (sd 462) | In supine position : 1351mL/min (sd 345)	NA	In sitting position: 9.0 mL/beat (sd 2.6) | In supine position: 9.2 mL/beat (sd 1.6)	NA	In sitting position: 70% (sd 15) | In supine position: 63% (sd 13)	NA	In sitting position: 72% (sd 15) | In supine position: 61% (sd 14)	NA	In sitting position: 85% (sd 12) | In supine position: NA	NA	NA	Ventilatory causes could not account for the reduced exercise capacity. Our findings on positional effects on SV and exercise capacity in PE support this hypothesis. During supine exercise the patients were consistently able to further increase SV	CPET protocol: incremental with 25W/min steps.
Castille et al.	1982	Symptomatic pectus deformities of the chest	USA	We studied exercise tolerance and pulmonary mechanics in patients with symptomatic and asymptomatic pectus deformities.	Cross-sectional study	NA	8 participants (7 with PEx and 1 with pectus carinatum) | age: range= 9 - 22 yo	NA	CPET on cycloergometer	Blood gas analysis	Spirometry	VO2 in L/min	NA	Continue measure of arterial gas	TLC in %	NA	range = 0.79L/min - 2.32L/min	NA	NA	Normal. No objectif data were reported	NA	NA	NA	NA	79%	range = 2.56 to 6.87L	The arterial oxygen tension, arterial carbon dioxide tension, pH, arterial lactate, plasma bicarbonate, alveolar-arterial oxygen difference, minute ventilation, ratio of physiologic dead space to tidal volume, and respiratory exchange ratio all display normal [illegible]	Although we did not measure chest wall compliance, we suspect that increased elastic loads imposed by the rib cage deformity may be responsible for the increased oxygen uptake in our symptomatic patients. « The performance [illegible]	CPET protocole : incremental with 4min steps.
Bevegard	1962	Postural Circulatory Changes at Rest and during Exercise in Patients with Funnel Chest, with Special Reference to Factors Affecting the Stroke Volume	Sweden	The purpose of the present investigation has been to find the hernodynamic explanation for the influence of body position on the physical working capacity in cases with funnel chest.	Cross-sectional study	NA	Age: range= 15 - 63 | 16 participants (6 women and 10 men)	NA	CPET on cycloergometer	Blood gas analysis and hemoglobin concentration	Spirometry	Physical working capacity in kpm/min at 170bpm heart rate	NA	Measures of blood gas and Hb at rest and two times during exercise	FVC in L, TLC in L	NA	In sitting position : From 220kpm/min to 1 110 kpm/min | In supine position : from 380 kpm/min to 1 090 [illegible]	NA	NA	SpO2: 93 - 99% | Hb (ing/100mL): 11.6 - 17.3 | Mixed veinous saturation in sitting position: [illegible]	NA	3.91 - 8.17L	NA	NA	NA	3.91 to 9.5L	NA	NA	CPET protocol: The PWC, is defined as the work intensity in kpm per minute, which the subject could perform at a pulse rate of 170 beats per minute. The work load was increased stepwise every 6 min. until a pulse rate of about 170 was obtained.
