# Supplementary material for: Exercise impairment in patients with pectus excavatum? A scoping review of evidence and role of arterial content change during effort
Source: Physiol Rep. 2026 Jul 6;14(13):e71005. doi: 10.14814/phy2.71005 (PMC13338107; doi:10.14814/phy2.71005)
Supplement: Supplementary file 3 — Data S3. [file PHY2-14-e71005-s001.pdf]

JBI critical appraisal tool for analytical cross-sectional studies (n = 11)											
Item	Bevegard	Castille et al.	Cavestri et	Ghory et al.	Malek et al.	Oleksak et al.	Ravanbakhsh	Sanjurjo et	Satur et al.	Swanson et	Zens et al.
Were the criteria for inclusion in the sample clearly	no	no	yes	yes	yes	yes	yes	yes	yes	yes	yes
Were the study subjects and the setting described	yes	yes	yes	yes	yes	yes	yes	yes	yes	yes	yes
Was the exposure measured in a valid and reliable	yes	yes	yes	unclear	yes	yes	yes	unclear	yes	yes	yes
Were objective, standard criteria used for	yes	yes	yes	yes	yes	yes	yes	yes	yes	yes	yes
Were confounding factors identified?	no	no	yes	yes	yes	yes	yes	yes	yes	yes	yes
Were strategies to deal with confounding factors	no	no	no	yes	yes	yes	yes	yes	yes	yes	yes
Were the outcomes measured in a valid and reliable	yes	yes	yes	yes	yes	yes	yes	unclear	yes	yes	yes
Was appropriate statistical analysis used?	unclear	no	yes	yes	yes	yes	yes	yes	yes	yes	yes
Overall appraisal	4/8	4/8	7/8	7/8	8/8	8/8	8/8	6/8	8/8	8/8	8/8
Risk categorization	high	high	low	low	low	low	low	moderate	low	low	low

Legend

Legend colour	
green	yes
red	no
orange	unclear
blue	not applicable

Risk categorization
low = 80-100%
moderate = 50 - 80%
high = 50% and less

Barker TH, Hasanoff S, Aromataris E, Stone JC, Leonardi-Bee J, Sears K, et al. The revised JBI critical appraisal tool for the assessment of risk of bias for analytical cross-sectional studies. JBI Evid Synth. 2026;24(3):401-8.

JBI Critical Appraisal Checklist for cohort studies (n = 16)																
Item	Abu-Tair et	Borowitz et	Cahill et al.	Das et al.	Dupuis et al.	Eldredge et	Jaroszewski	Kelly et al.	Maagaard et	Morshuis et	Neviere et al.	O'keef et al.	Sigalet et al.	Tang et al.	Udholm et al.	Zhao et ak.
1. Were the two groups similar and recruited from	yes	NA	yes	NA	NA	NA	yes	yes	yes	NA	yes	NA	NA	yes	NA	yes
2. Were the exposures measured similarly to assign	yes	NA	yes	NA	NA	NA	NA	NA	yes	NA	NA	NA	NA	yes	NA	yes
3. Was the exposure measured in a valid and	yes	yes	yes	yes	yes	yes	yes	yes	yes	yes	yes	yes	yes	yes	yes	yes
4. Were confounding factors identified?	yes	yes	no	yes	yes	yes	yes	yes	yes	no	yes	yes	yes	yes	yes	yes
5. Were strategies to deal with confounding factors	yes	unclear	no	unclear	no	unclear	yes	yes	yes	no	yes	yes	yes	yes	yes	no
6. Were the groups/participants free of the	NA	NA	NA	NA	NA	NA	NA	NA	no	NA	no	no	no	no	NA	no
7. Were the outcomes measured in a valid and	yes	yes	yes	yes	yes	yes	yes	yes	yes	yes	yes	yes	yes	yes	yes	yes
8. Was the follow up time reported and sufficient to	NA	yes	yes	yes	NA	yes	yes	yes	yes	yes	yes	yes	yes	yes	yes	NA
9. Was follow up complete, and if not, were the	NA	no	unclear	unclear	no	yes	yes	yes	yes	unclear	no	yes	unclear	unclear	no	NA
10. Were strategies to address incomplete follow up	NA	no	no	no	no	no	no	no	no	no	no	no	no	no	unclear	NA
11. Was appropriate statistical analysis used?	yes	yes	yes	yes	yes	yes	yes	yes	yes	yes	yes	yes	yes	yes	yes	yes
Overall appraisal	7/11	5/11	6/11	5/11	4/11	6/11	8/11	8/11	9/11	4/11	7/11	7/11	6/11	8/11	6/11	6/11
Risk categorization	moderate	high	moderate	high	high	moderate	moderate	moderate	low	high	moderate	moderate	moderate	moderate	moderate	moderate

Legend

Legend colour	
green	yes
red	no
orange	unclear
blue	not applicable (NA)

Risk categorization
low = 80-100%
moderate = 50 - 80%
high = 50% and less

Moola S, Munn Z, Tufanaru C, Aromataris E, Sears K, Sfetcu R, Currie M, Qureshi R, Mattis P, Lisy K, Mu P-F. Chapter 7: Systematic reviews of etiology and risk . In: Aromataris E, Munn Z (Editors). JBI Manual for Evidence Synthesis. JBI, 2020.

JBI Critical Appraisal Checklist for quasi-experimental studies (n = 4)				
Item	Al-Assiri	ller and Lough	Quigley et al.	Wynn et al.
1. Is it clear in the study what is the "cause" and	yes	yes	yes	yes
2. Was there a control group?	unclear	yes	unclear	yes
3. Were participants included in any comparisons	yes	yes	yes	yes
4. Were the participants included in any	yes	yes	yes	yes
5. Were there multiple measurements of the	yes	yes	yes	yes
6. Were the outcomes of participants included in	unclear	unclear	unclear	unclear
7. Were outcomes measured in a reliable way?	yes	yes	yes	yes
8. Was follow-up complete and if not, were	yes	yes	yes	yes
9. Was appropriate statistical analysis used?	yes	yes	yes	yes

**Overall appraisal**	**7/9**	**8/9**	**7/9**	**8/9**
**Risk categorization**	**moderate**	**low**	**moderate**	**low**

Legend

Legend	Risk categorization
yes	low = 80-100%
no	moderate = 50 - 80%
unclear	high = 50% and less
not applicable (NA)	

Barker TH, Habibi N, Aromataris E, Stone JC, Leonardi-Bee J, Sears K, et al. The revised JBI critical appraisal tool for the assessment of risk of
