# Supplementary figures and images for: Exercise impairment in patients with pectus excavatum? A scoping review of evidence and role of arterial content change during effort
Source: Physiol Rep. 2026 Jul 6;14(13):e71005. doi: 10.14814/phy2.71005 (PMC13338107; doi:10.14814/phy2.71005)

## Hypothesis

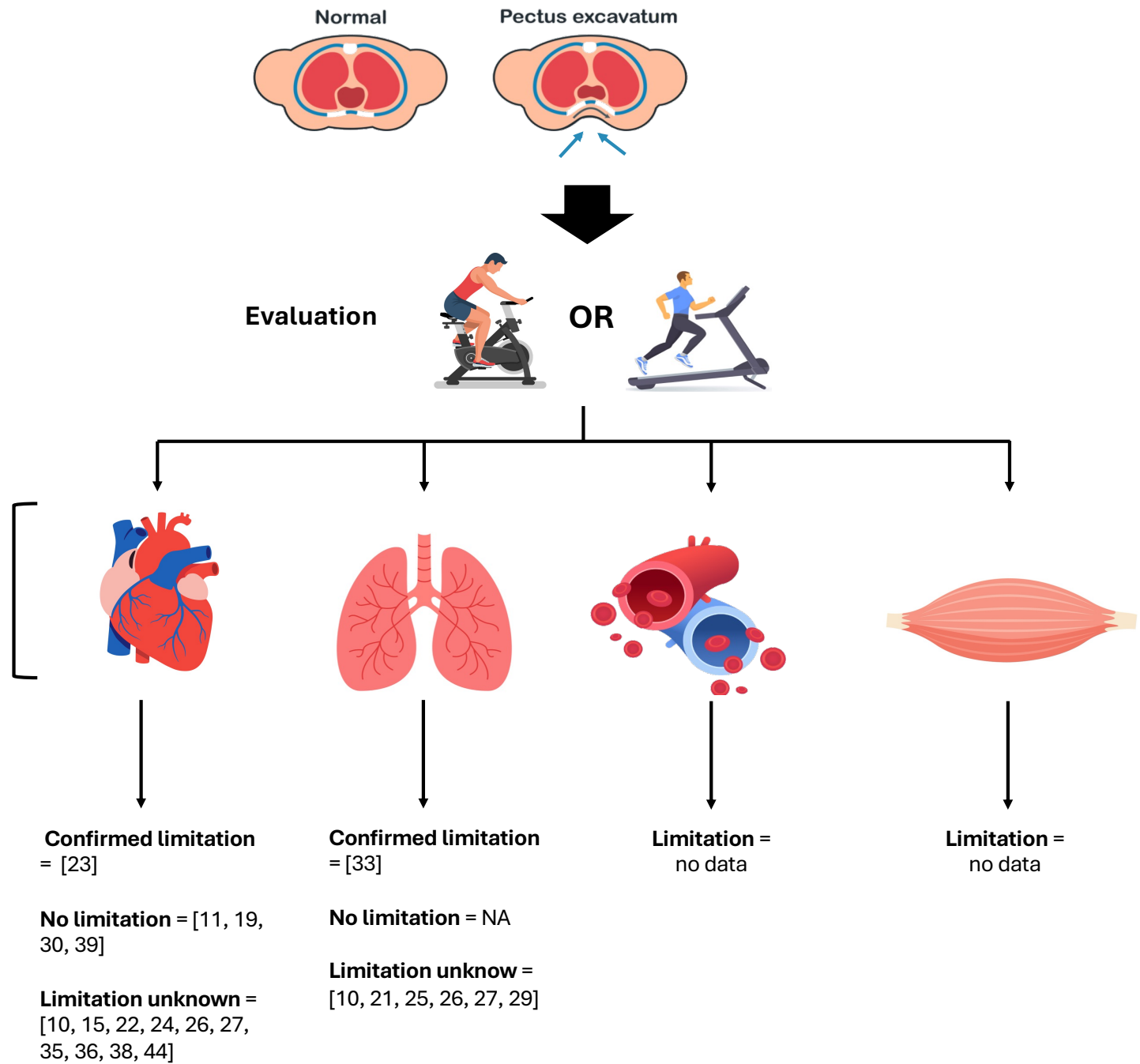

Supplement: Supplementary file 4 — Data S4. [file PHY2-14-e71005-s002.pdf]
