# Supplementary material for: Exercise impairment in patients with pectus excavatum? A scoping review of evidence and role of arterial content change during effort
Source: Physiol Rep. 2026 Jul 6;14(13):e71005. doi: 10.14814/phy2.71005 (PMC13338107; doi:10.14814/phy2.71005)
Supplement: Supplementary file 5 — Data S5. [file PHY2-14-e71005-s006.pdf]

**SUPPLEMENTARY MATERIAL 5**

*Pathophysiological hypothesis*

*This supplementary file provides further elaboration on the results and the pathophysiological hypotheses discussed by the authors of the included studies. It has its own bibliography.*

## PATHOPHYSIOLOGICAL HYPOTHESIS PROPOSED BY AUTHORS

Extracted physiopathological hypothesis proposed by authors are presented in Table A.

Authors of 21 out of the 31 included articles discussed one or more pathophysiological hypotheses to explain the reduced exercise capacity observed in some patients with PEx. Among them, 13 articles mentioned the reduction of stroke volume (SV) due to right heart compression by the sternum as a hypothesis [1-13]. Nevertheless, only the articles by Abu-Tair et al., Zen et al. and Sigalet et al. reported measuring SV [3, 8, 9]. Four articles mentioned an increased respiratory effort due to the thoracic deformity, leading to higher energy expenditure without improved performance during exercise [8, 14-16]. Nevertheless, only the articles by Cavestri and colleagues, as well as the article by Sigalet and colleagues, reported objective measurements of deformity and respiratory outcomes [8, 16]. Three articles mentioned a reduced static ventilatory reserve (SVR) that leads to lower tidal volume and maximal ventilation, along with a potentially increased respiratory workload [7, 17, 18]. Objective respiratory outcome measures were presented in the articles by Morshuis and colleagues as well as by O'Keef and colleagues [7, 18]. One article reported an objective measure of decreased both right and left systolic function [9] and suggested it as a cause of exercise impairment. In this article, cardiac function was assessed throughout right ventricular ejection fraction, left ventricular ejection fraction, end-diastolic volume, end-systolic volume, SV, ventricular mass, body surface area–indexed values. One article reported an improvement in SV measured using Doppler when lied down, suggesting a role for body posture [19]. One article rejected the hypothesis that reduced exercise capacity is merely due to deconditioning or lack of motivation [20] but suggested no other explanation. Authors based their hypothesis on the observation that patients achieved maximal effort during CPET, evidenced by high RER and significant acid–base shifts, while still exhibiting multiple objective abnormalities in cardiopulmonary parameters, indicating true physiological dysfunction rather than simple deconditioning. Finally, one article reported impaired venous return to the right atrium and consequently decreased cardiac output, attributed to thoracic rigidity [17]. Figure 1 represents repartition of pathophysiological hypothesis proposed by authors regarding physiological systems involved in exercise capacity.

## Table A. Physiopathological hypothesis

| Article | Physiopathological hypothesis reported by authors | Targeted system | Comment |
|---|---|---|---|
| Abu-Tair et al. (2018) | Reduced stroke volumes due to a decrease in retrosternal space, leading to impaired diastolic function of the right ventricle. In this case, the mechanism is presumably compensated by an increase in heart rate at the anaerobic threshold. | Cardiac function | $O_2P$ was evaluated, but the authors did not specify how it was evaluated. In addition, the authors did not assess A-V $O_2$ diff. |
| Al-Assiri (2009) | NA | NA | NA |
| Bevegard (1962) | NA | NA | NA |
| Borowitz et al. (2003) | NA | NA | NA |
| Cahill et al. (1984) | Patients with restrictive pulmonary disorders, such as severe pectus excavatum deformity, may not be able to increase ventilation because of restriction in chest wall mechanics. | Pulmonary function | Authors measured the TLC in patients with PEx before and after surgery. The severity of the PEx deformity was not measured. |
| Castile et al. (1982) | Although we did not measure chest wall compliance, we suspect that increased elastic loads imposed by the rib cage deformity may be responsible for the increased oxygen uptake in our symptomatic patients. The performance of equal exertional tasks by symptomatic patients relative to their peers would require an additional expenditure of energy proportional to the increased work of breathing. | Pulmonary function | Authors did not measure chest wall compliance. |
| Cavestri et al. (2010) | Le déconditionnement pourrait expliquer en partie la limitation à l'exercice. Cependant, Malek et al. ont montré qu'il existait une diminution de l'aptitude aérobie chez des patients atteints de PEx pratiquant une activité sportive régulière (trois heures par semaine en moyenne), ce qui laisse supposer que la déformation en elle-même est susceptible d'induire une diminution de l'aptitude aérobie. Nos données viennent confirmer cette hypothèse. | Pulmonary function | Authors have measured physical activity level and frequency. Authors also measured $O_2P$ and A-a$DO_2$. Authors also measured the Haller Index as well as the thoracic oppression. |
| Das et al. (2019) | The improvement of maximum oxygen consumption in our study after surgical repair appears to be due to increase in stroke volume during exercise, and our results are similar to other published studies. | Cardiac function | Authors reported pre- and post-operative data for maximum oxygen consumption. Authors measured $O_2P$, but did not measure A-V $O_2$ diff. |

| | | | |
|---|---|---|---|
| Dupuis et al. (2024) | Other studies suggest PEx may result in a decreased capacity to increase systolic ejection volume during exercise, due to heart compression. Compression of the right heart cavity could contribute to mitral valve prolapse according to some studies, an hypothesis supported by a higher prevalence of valvular disease in patients with PEx. | Cardiac function | This hypothesis is based on previous studies. Authors reported that $O_2P$ was normal and precise that the evaluation of the cardiac function was minimal. Authors did not measure A-V $O_2$ diff. |
| Eldredge et al. (2025) | The improvement in the stroke volume response may be secondary to the anatomic relief of cardiac compression due to the PEx deformity following MIRPE. | Cardiac function | Authors measured $O_2P$ and heart rate before and after a surgery procedure. They did not measured A-V $O_2$ diff. |
| Ghory et al. (1989) | NA | NA | NA |
| Haller and Loughlin (2000) | The most likely reason for the increased oxygen pulse is an increased stroke volume due to relief of cardiac compression by the sternal deformity. | Cardiac function | Authors measured $O_2P$, but they did not measured A-V $O_2$ diff. |
| Jaroszewski et al. (2022) | NA | NA | NA |
| Kelly et al. (2013) | We measured the oxygen pulse during peak or maximum exercise and have interpreted this increase in $O_2P$ to reflect an increase in stroke volume. | Cardiac function | Authors measured $O_2P$ but they did not measured A-V $O_2$ diff. |
| Maagaard et al. (2013) | Further accentuates the association between postoperative improved function and an increase in cardiac (stroke) volume during exercise. | Cardiac function | Authors measured the stroke volume using the maximum stroke index. They did not measure $O_2P$ nor A-V $O_2$ diff. |
| Malek et al. (2003) | NA | NA | NA |
| Morshuis et al. (1994) | Castile, Staats, and Westbrook found that V02 during exercise exceeded predicted values at greater work loads in five patients with symptomatic pectus excavatum, whereas two patients without symptoms did not show the same process. These investigators suggested that these findings in patients with symptomatic deformities were caused by decreased chest wall compliance and an increased work of breathing. Because the oxygen uptake at rest was unchanged, the significant increase in VO2 after operation together with increased acidification at exercise may suggest that more muscle activity is needed at maximal exercise that does not contribute to external work load, which may indicate an increased activity of the ventilatory musculature. | Pulmonary function | Authors did not measure chest wall compliance but measured ventilation. |

| | | | |
|---|---|---|---|
| Neviere et al. (2011) | This is in line with previous studies showing significant decreases in O2 uptake and O2 pulse in PE patients, which was not explained by physical deconditioning. These findings suggest that, in PE patients, cardiac filling and cardiac output might be limited by the result of compression by the displaced sternum on the right heart chambers, thereby limiting increase of stroke volume. At exercise, these changes might impede further the heart's ability to increase the stroke volume, to meet increased metabolic demands. These findings suggest that PE repair could improve cardiac filling and cardiac output at maximal workload as a result of the chest's anteroposterior diameter increase. | Cardiac function | Authors measured left and right ventricular as well as ventricular ejection fraction, peak early filling mitral flow velocity, DLCO, $O_2$P and A-a$DO_2$. |
| O'Keefe et al. (2013) | We believe in part these results highlight the limitations of extrapolating cardiopulmonary assessment done at rest to the physiologic effects with exercise. The subjective improvement after repair is classically described by the patients as an improvement in maximum exercise capacity. Pectus excavatum creates a fixed restriction in the chest volume which affects the heart, decreasing stroke volume and hence cardiac output, especially at high load. | Cardiac function and pulmonary function | Authors measured $O_2$P, heart rate and cardiac index, but they did not measured A-V $O_2$ diff. Authors measured respiratory outcome measures. |
| Oleksak et al. (2022) | According to Malek et al. (2006) sternal compression reduces the sternum volume, leading to a reduction in maximal oxygen consumption during exercise, exercise tolerance, tidal volume and vital capacity, all of which reduce body endurance and cause dyspnoea and compensatory tachypnoea during exercise. Reduced oxygen supply to the working muscle as a consequence of reduced venous return to the right atrium also contributes to the reduced physical fitness of patients with PE. | Cardiac function and pulmonary function | Authors measured $O_2$P and HR. They did not measure sternum volume neither A-V $O_2$ diff. Authors measured the breathing reserve. |
| Quigley et al. (1996) | The most likely reason for the increased $O_2$P postoperatively is an increased stroke volume because of relief of cardiac compression by the sternal deformity. | Cardiac function | Authors did not measure stroke volume. Authors measured $O_2$P, but they did not measure A-V $O_2$ diff. |
| Ravanbakhsh et al. (2022) | NA | NA | NA |
| Sanjurjo et al. (2024) | NA | NA | NA |
| Satur et al. (2021) | The results contradict the view that patients with PE are 'deconditioned', more bluntly described, 'lazy' with a lack of physical motivation. The mechanistic cause of exercise dysfunction and subsequent improvement following surgical treatment however, remain unproven. | | Authors did not measure physical motivation. |

| | | | |
|---|---|---|---|
| Sigalet et al. (2007) | It is due to a combination of improved efficiency of ventilation, and increased stroke volume (and secondarily cardiac output) at exercise. The perceived shortness of breath with intense exercise, which patients describe preoperatively is likely due to the reduced stroke volume at load caused by the pectus compression. This suggests that with correction of the pectus defect, there is an increase in the efficiency of the chest wall. This would free up oxygen and energy available for general musculoskeletal use. | Cardiac function and pulmonary function | Authors did measure pectus severity, stroke volume as well as cardiac output and $O_2P$, but did not report A-V $O_2$ diff. |
| Swanson et al. (2012) | One hypothesis is that the right ventricle may experience compression earlier and with greater magnitude compared with the lungs, with resultant decreased right ventricular filling, because of its immediately substernal location and smaller relative volume. | Cardiac function | No measure of the compression or the ventricular filling. Authors measured $O_2P$, but did not measure A-V $O_2$ diff. |
| Tang et al. (2012) | NA | NA | NA |
| Udholm et al. (2016) | NA | NA | NA |
| Wynn et al. (1990) | NA | NA | NA |
| Zens et al. (2022) | Despite compression of the right side of the heart, LV function was also affected, with LVEF <0.55 in 22% of patients and an LVEF Z-score of < 2 in 18% of patients, highlighting the potential for pectus excavatum to have a detrimental effect on biventricular cardiac function. | Cardiac function | Authors measured pectus deformation, cardiac deformation as well as left and right ventricular ejection fraction and $O_2P$ but did not measure A-V $O_2$ diff. |
| Zhao et al. (2000) | Ventilatory causes could not account for the reduced exercise capacity. Our findings on positional effects on SV and exercise capacity in PE support this hypothesis. During supine exercise the patients were consistently able to further increase SV (and oxygen-pulse) to a level that on average was not different from that of the control subjects. In contrast, SV in the control subjects was not significantly higher, and $VO_2max$ was actually lower in the supine position, indicating that the positional advantage was unique to the patients with PE. | Cardiac function | Authors measured stroke volume with Doppler at rest and during effort, DLCO and $O_2P$ but did not measure A-V $O_2$ diff. |

PEx = pectus excavatum, A-a$DO_2max$ = alveolar arterial oxygen difference at maximal exercise, DLCO = diffusing capacity of the lung for carbon monoxide, A-V $O_2$ diff = arteriovenous oxygen difference, $O_2P$ = Oxygen pressure

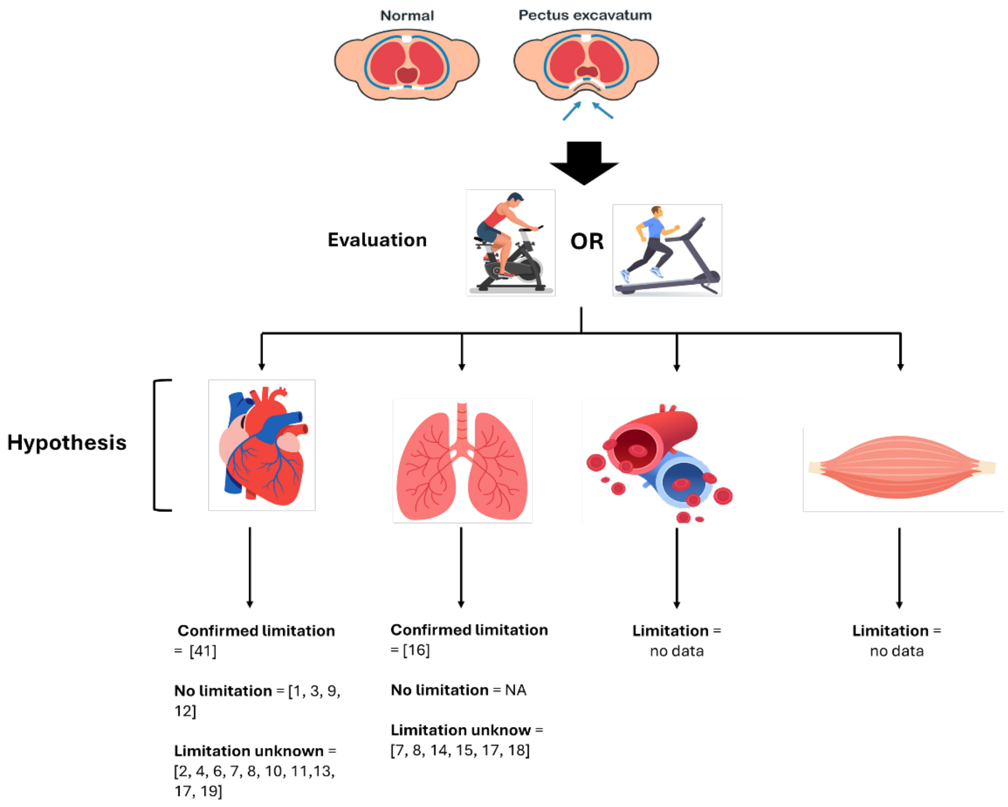


**Figure A.** Repartition of pathophysiological hypothesis regarding physiological systems.

## DISCUSSION OF PATHOPHYSIOLOGICAL HYPOTHESIS

Surprisingly, four articles [1, 3, 9, 12] proposed pathophysiological hypotheses to explain exercise impairment, despite their cohorts not demonstrating such impairment based on the 85% $VO_2$max threshold discussed in the main text (see 4.1. section). Even more notably, Abu-Tair et al. [3] did not report reduced exercise capacity, but rather an abnormal SV during exercise, which they still used as a pathophysiological explanation. Among the 31 articles included in this review, 12 proposed that reduced SV due to right heart compression by the sternum may underlie exercise limitations. However, only three of these articles [3, 8, 9] directly measured SV. The other ten relied on the findings of these three articles to support their hypotheses. Interestingly, Sonaglioni et al. [21] published in 2023 a systematic review focusing on the impact of PEx on cardiac mechanics and function, and they cited five articles as evidence for SV reduction in patients with PEx. Two of them were articles from Malek et al. [22] and Haller and Loughlin [10] included in the present scoping review, and might reported results provided by Abu-Tair et al. [3], Sigalet et al. [8] or Zen et al. [9] because they did not directly measure SV. The systematic review subsequently cited a case study [23] and another study that reported an increase in stroke volume following PEx repair, although neither clearly confirmed a reduced stroke volume prior to surgery [24]. Therefore, we may observe that this hypothesis provided by most of the authors focusing on PEx seems to be founded on only three articles. This is understandable, as the idea of cardiac compression is intuitive and relatively easy to conceptualize, whereas directly measuring SV requires sophisticated and often costly methods. However, this well-known hypothesis might not motivate further

articles focusing on other pathophysiological mechanisms that may cause exercise impairment, even if this hypothesis is not strongly confirmed. Of the 12 articles proposing the presence of a reduced SV as a cause of impaired exercise capacity, seven based their hypothesis on $O_2$Pmax values [2, 3, 4, 6, 8, 10, 11]. $O_2$Pmax is defined as $VO_2$max divided by HR at $VO_2$max, which is equals to SV multiplied by the A-V $O_2$ diff, both at $VO_2$max. However, this reasoning neglects the possibility that a reduced $O_2$Pmax may also result from a decreased A-V $O_2$ diff, independently of SV. A reduction in A-V $O_2$ diff can occur without any cardiac dysfunction and has been documented in conditions such as fibromyalgia [25], mitochondrial disorders [26], anemia [27], or even in heart failure with preserved ejection fraction [28]. In mitochondrial or metabolic diseases, impaired oxidative phosphorylation at the muscular level reduces oxygen extraction despite normal or elevated cardiac output [26]. Similarly, in severe anemia, arterial oxygen content is decreased, limiting $VO_2$ independently of SV [27]. While speculative, these mechanisms emphasize that a low $O_2$Pmax should not automatically be attributed to reduced cardiac output or SV. Additionally, severe physical deconditioning, proposed by Cavestri et al. [16] but dismissed by Satur et al. [20] and Malek et al. [22] to explain exercise intolerance in patients with PEx, can also lead to a low $O_2$Pmax due to peripheral inefficiency in oxygen extraction. This further highlights the need for caution when interpreting $O_2$Pmax in the absence of direct SV measurements. The repeated reliance on this single parameter to support the cardiac compression hypothesis appears reductionist and underscores the need for a broader physiological assessment. Such an assessment should include hemoglobin concentration, muscle oxidative capacity, and non-invasive estimates of A-V $O_2$ diff, such as near-infrared spectroscopy or rebreathing techniques, as used by Sigalet et al. [8]. This opens the way to a new pathophysiological hypothesis, never previously proposed in the literature reviewed, based on hemoglobin and/or oxygen content in blood changes during exercise.

We hypothesize that in some patients with PEx, exercise-intolerance induced impairment of oxygen delivery may result from a right-to-left cardiac shunt via a patent foramen ovale (PFO). This has been documented in two case reports [29, 30], in another study reporting a 17% prevalence [31], and in 28% of the cohort studied by Cavestri et al. [16]. This prevalence appears higher than in the general population, where PFO is present in 13–25% of adults [32, 33]. Furthermore, 61% of PFOs in patients with PEx reportedly close spontaneously after surgical correction [30, 31], suggesting that the thoracic deformity may prevent natural closure in some cases. Such a mechanism would allow desaturated venous blood to bypass pulmonary circulation and pass from the right (higher pressure) to the left (lower pressure) atrium. As a result, part of the systemic circulation would carry deoxygenated blood, leading to arterial hypoxemia. A case report described a $SpO_2$ drop from 97% to 92% during exercise in a female with PEx and PFO [30]. The association between PEx and PFO may be accounted for by atrial septal distortion induced by the thoracic deformity, together with altered cardiac chamber geometry, which could promote venous return via the inferior vena cava directed toward a potential PFO [30]. If the 28% prevalence reported by Cavestri et al. were generalizable to the broader PEx population, it could explain why some patients experience exercise-induced desaturation or more broadly exercise impairment. Moreover, given the intermittent nature of such shunting—especially during physical effort or when right atrial pressure increases, as during Valsalva maneuvers—this mechanism might be missed during

routine resting evaluations. Yet, this scoping review revealed no additional data on PFO in PEx beyond the article by Cavestri et al., exposing a significant knowledge gap. Future studies should investigate the prevalence and physiological consequences of PFO in larger and well-characterized PEx cohorts.

Another potential mechanism supporting this hypothesis involves exercise-induced pulmonary hypertension caused by pulmonary artery compression. A case report described post-stenotic dilatation of a pulmonary artery compressed within the reduced anteroposterior thoracic space in PEx [34]. This suggests that even moderate pulmonary artery compression might not raise pulmonary pressure at rest but could do so during exercise, as cardiac output increases. Right pulmonary artery compression has been documented in case-control studies as a cause of hypoxemia, such as in patients with mediastinal tumors [35] or during surgery due to sternal retractors [36]. This rise in right ventricular and atrial pressures could further promote right-to-left shunting in patients with PFO, resulting in systemic hypoxemia as previously hypothesized. However, to our knowledge, no study has investigated this hypothesis in any patient population.

Conceptually, it is likely that not all patients with PEx have limitations. First, in relatively young subjects, compensatory mechanisms may lead to reasonable tolerance [37], but these compensatory mechanisms in young adults may lead to fatigue and could explain the apparent worsening of exercise capacity in non-operated adult patients. Second, when limitations are present, the underlying mechanisms may vary among patients and depend on individual anatomical differences, the severity of the thoracic deformity, and also the type of deformity [38-40], such as the vertical extension.

**REFERENCES:**


1. Dupuis, M., Daussy, L., Noel-Savina, E., Dahan, M., Didier, A., Chavoin, J., Guibert, N. Impact of pectus excavatum on pulmonary function and exercise capacity in patients treated with 3D custom-made silicone implants. *Annales de Chirurgie Plastique Esthétique*, 2024;69(1)
2. Neviere, R., Montaigne, D., Benhamed, L., Catto, M., Edme, J. L., Matran, R., & Wurtz, A. Cardiopulmonary response following surgical repair of pectus excavatum in adult patients. *European Journal of Cardio-Thoracic Surgery*, 2011;40(2)
3. Abu-Tair, T., Turial, S., Hess, M., M., C., Staatz, G., Lollert, A., Kampmann, C. Impact of Pectus Excavatum on Cardiopulmonary Function. *The Annals of Thoracic Surgery*, 2018;105(2)
4. Das BB, Recto MR, Yeh T. Improvement of cardiopulmonary function after minimally invasive surgical repair of pectus excavatum (Nuss procedure) in children. *Annals of Pediatric Cardiology*, 2019;12(2)
5. Scott, R., Sabati, A., Ochoa, B., Viswanath, V., Khoury, E., Rassam, K., J., D., Lee, J., McMahon, L., M., D., E., B. Cardiopulmonary Impact of the Minimally Invasive Repair of Pectus Excavatum in Pediatric Patients: A Prospective Pilot Study. Journal of Pediatric Surgery, 2025;60(4)

6. Kelly, R. E., Jr, Mellins, R. B., Shamberger, R. C., Mitchell, K. K., Lawson, M. L., Oldham, K. T., Azizkhan, R. G., Hebra, A. V., Nuss, D., Goretsky, M. J., Sharp, R. J., Holcomb, G. W., 3rd, Shim, W. K., Megison, S. M., Moss, R. L., Fecteau, A. H., Colombani, P. M., Cooper, D., Bagley, T., Quinn, A., … Paulson, J. F. Multicenter study of pectus excavatum, final report: complications, static/exercise pulmonary function, and anatomic outcomes. Journal of the American College of Surgeons, 2013;217(6)

7. O'Keefe, J., Byrne, R., Montgomery, M., Harder, J., Roberts, D., L., D. Longer term effects of closed repair of pectus excavatum on cardiopulmonary status. *Journal of Pediatric Surgery*, 2013;48(5)

8. Sigalet L.D., Montgomery, M., Harder, J., Wong, V., Kravarusic, D., Alassiri, A. Long term cardiopulmonary effects of closed repair of pectus excavatum. *Pediatric Surgery International*, 2007;23(5)

9. Zens, T. J., Casar Berazaluce, A. M., Jenkins, T. M., Hardie, W., Alsaied, T., Tretter, J. T., Moore, R., Foster, K., Fleck, R. J., Hanke, R. E., Colvin, B. E., Garrison, A. P., Kraemer, A., Crotty, E., Taylor, M., Garcia, V. F., & Brown, R. L. The Severity of Pectus Excavatum Defect Is Associated With Impaired Cardiopulmonary Function. The Annals of Thoracic Surgery, 2022;114(3)

10. Haller, J.A., & Loughlin, G.M. Cardiorespiratory function is significantly improved following corrective surgery for severe pectus excavatum. Proposed treatment guidelines. *The Journal of Cardiovascular Surgery*, 2000;41(1)

11. Quigley, P. M., Haller Jr, J. A., Jelus, K. L., Loughlin, G. M., & Marcus, C. L. Cardiorespiratory function before and after corrective surgery in pectus excavatum. *The Journal of Pediatrics*, 1996;128(5)

12. Swanson, J. W., Avansino, J. R., Phillips, G. S., Yung, D., Whitlock, K. B., Redding, G. J., & Sawin, R. S. Correlating Haller Index and cardiopulmonary disease in pectus excavatum. *American Journal of Surgery*, 2012;203(5)

13. Maagaard, M., Tang, M., Ringgaard, S., Henrik, H., Frøkiær, J., Haubuf, M., K., H., E., V. Normalized Cardiopulmonary Exercise Function in Patients With Pectus Excavatum Three Years After Operation. *The Annals of Thoracic Surgery*, 2013;96(1)

14. Cahill, J. L., Lees, G. M., & Robertson, H. T. A summary of preoperative and postoperative cardiorespiratory performance in patients undergoing pectus excavatum and carinatum repair. *Journal of Pediatric Surgery*, 1984;19(4)

15. Castile, R. G., Staats, B. A., & Westbrook, P. R. Symptomatic pectus deformities of the chest. *American Review of Respiratory Disease*, 1982;126(3)

16. Cavestri, B., Wurtz, A., Bart, F., Nevière, R., Aguilaniu, B., Wallaert, B. Exploration fonctionnelle à l'exercice des patients présentant un pectus excavatum. *Revue des Maladies Respiratoires*, 2010;27(7)

17. Oleksak, F., Spakova, B., Durdikova, A., Durdik, P., Kralova, T., Igaz, M., ... & Murgas, D. Correlation of anthropometric index and cardiopulmonary exercise testing in children with pectus excavatum. *Respiratory Physiology & Neurobiology*, 2022;296

18. Morshuis, W. J., Folgering, H. T., Barentsz, J. O., Cox, A. L., van Lier, H. J., & Lacquet, L. K. Exercise cardiorespiratory function before and one year after operation for pectus excavatum. *The Journal of Thoracic and Cardiovascular Surgery*, 1994;107(6)

19. Zhao, L., S., M., Gaides, M., Ben-Dov, I. Why is exercise capacity reduced in subjects with pectus excavatum?. *The Journal of Pediatrics*, 2000;136(2)

20. M, C., Cliff, I., Watson, N. Can categorised values of maximal oxygen uptake discriminate patterns of exercise dysfunction in pectus excavatum: a prospective cohort study?. *BMJ Open Respiratory Research*, 2021;8(1)

21. Sonaglioni, A., Luigi, G., Trevisan, R., Lombardo, M., Grasso, E., Franco, G., Ambrosio, G. The influence of pectus excavatum on cardiac kinetics and function in otherwise healthy individuals: A systematic review. *International Journal of Cardiology*, 2023;381

22. Malek, M. H., Fonkalsrud, E. W., & Cooper, C. B. Ventilatory and cardiovascular responses to exercise in patients with pectus excavatum. *Chest*, 2003;124(3)

23. Jaroszewski, D., Steidley, E., Galindo, A., Arabia, F. Treating Heart Failure and Dyspnea in a 78-Year-Old Man With Surgical Correction of Pectus Excavatum. *The Annals of Thoracic Surgery*, 2009;88(3)

24. Kowalewski, J., Brocki, M., Dryjanski, T., Zolyński, K., Koktysz, R. Pectus excavatum: Increase of right ventricular systolic, diastolic, and stroke volumes after surgical repair. *The Journal of Thoracic and Cardiovascular Surgery*, 1999;118(1)

25. Lehto, T., Zetterman, T., Markkula, R., Arokoski, J., Tikkanen, H., Kalso, E., E., J. Cardiac output and arteriovenous oxygen difference contribute to lower peak oxygen uptake in patients with fibromyalgia. *BMC Musculoskeletal Disorders*, 2023;24(1)

26. Taivassalo, T. The spectrum of exercise tolerance in mitochondrial myopathies: a study of 40 patients. *Brain*, 2003;126(2)

27. Webb, K. L., Gorman, E. K., Morkeberg, O. H., Klassen, S. A., Regimbal, R. J., Wiggins, C. C., ... & Senefeld, J. W. The relationship between hemoglobin and V˙O2max: A systematic review and meta-analysis. *PLoS One*, 2023;18(10)

28. Naito, A., Obokata, M., Kagami, K., Harada, T., Sorimachi, H., Yuasa, N., Saito, Y., Kato, T., Wada, N., Adachi, T., Ishii, H. Contributions of anemia to exercise intolerance in heart failure with preserved ejection fraction–An exercise stress echocardiographic study. *IJC Heart & Vasculature*, 2023;48

29. Wallaert, B., Cavestri, B., Fournier, C., Nevière, R., Aguilaniu, B. Positional hyperventilation-induced hypoxaemia in pectus excavatum. *European Respiratory Journal*, 2006;28(1)

30. Tricard, J., Maltais, F., Rodès-Cabau, J., Conti, M. Pectus Excavatum, Patent Foramen Ovale, and Migraine in the Same Patient. *JACC: Case Reports*, 2025;30(22)

31. Wurtz, A., Rousse, N., Benhamed, L., Conti, M., Hysi, I., Pinçon, C., Nevière, R. Simplified open repair for anterior chest wall deformities. Analysis of results in 205 patients. *Orthopaedics & Traumatology: Surgery & Research*, 2012;98(3)

32. Kelly, T., Patrician, A., Bryant-Ekstrand, M., Brown, C., Gasho, C., G., H., N., R., Dawkins, T., Drane, A., Stembridge, M., Dragun, T., Barak, O., Spajić, B., Drviš, I., W., J., E., G., N., P., Dujić, Ž., T., A. High prevalence of patent foramen ovale in recreational to elite breath hold divers. *Journal of Science and Medicine in Sport*, 2022;25(7)

33. Purvis, J. A., Morgan, D. R., & Hughes, S. M. Prevalence of patent foramen ovale in a consecutive cohort of 261 patients undergoing routine. *The Ulster Medical Journal*, 2011;

34. D'Cruz, I., Shirwany, A., Gerlach, P. Pectus Excavatum Associated with Pulmonary Artery Dilatation. *Echocardiography*, 2004;21(1)

35. Takeda, S., Miyoshi, S., Omori, K., Okumura, M., Matsuda, H. Surgical rescue for life-threatening hypoxemia caused by a mediastinal tumor. *The Annals of Thoracic Surgery*, 1999;68(6)

36. Onwere, J. L., Spackman, T. N., & Click, R. L. Intraoperative hypoxemia from compression of the right pulmonary artery caused by a sternal retractor. *Anesthesia and Analgesia*, 2008;106(2)

37. Jaroszewski, D. E., & Fonkalsrud, E. W. Repair of pectus chest deformities in 320 adult patients: 21 year experience. *The Annals of Thoracic Surgery*, 2007;84(2)

38. Choi, J., Kyu, I., Tae, Y., Sun, W., Hyun, C. Classification of Pectus Excavatum According to Objective Parameters From Chest Computed Tomography. *The Annals of Thoracic Surgery*, 2016;102(6)

39. Zeineddine, R. M., Khedr, A. E., Farina, J. M., Odeh, N. B., Senjab, A., Jenkins, J. A., & Jaroszewski, D. E. Pectus Arcuatum Definitions, Diagnostics, and Surgical Guidelines: A Systematic Review. *JAMA Surgery*, 2025;10.1001/jamasurg.2025.2365. Advance online publication

40. Janssen, N., Coorens, N. A., Franssen, A. J. P. M., Daemen, J. H. T., Michels, I. L., Hulsewé, K. W. E., Vissers, Y. L. J., & de Loos, E. R. Pectus excavatum and carinatum: a narrative review of epidemiology, etiopathogenesis, clinical features, and classification. *Journal of Thoracic Disease*, 2024;16(2)

41. Eldredge, R. S., Sabati, A., Ochoa, B., Viswanath, V., Khoury, E., Rassam, K., ... & Padilla, B. E. (2025). Cardiopulmonary impact of the minimally invasive repair of pectus excavatum in pediatric patients: a prospective pilot study. *Journal of Pediatric Surgery*, 60(4), 162177.
